# An Unbiased Cell Morphology–Based Screen for New, Biologically Active Small Molecules

**DOI:** 10.1371/journal.pbio.0030128

**Published:** 2005-04-05

**Authors:** Masahiro Tanaka, Raynard Bateman, Daniel Rauh, Eugeni Vaisberg, Shyam Ramachandani, Chao Zhang, Kirk C Hansen, Alma L Burlingame, Jay K Trautman, Kevan M Shokat, Cynthia L Adams

**Affiliations:** **1**Department of Cellular and Molecular Pharmacology, University of CaliforniaSan Francisco, CaliforniaUnited States of America; **2**Cytokinetics Inc., South San FranciscoCaliforniaUnited States of America; **3**Department of Pharmaceutical Chemistry, Mass Spectrometry FacilityUniversity of California, San Francisco, CaliforniaUnited States of America; National Cancer InstituteUnited States of America

## Abstract

We have implemented an unbiased cell morphology–based screen to identify small-molecule modulators of cellular processes using the Cytometrix (TM) automated imaging and analysis system. This assay format provides unbiased analysis of morphological effects induced by small molecules by capturing phenotypic readouts of most known classes of pharmacological agents and has the potential to read out pathways for which little is known. Four human-cancer cell lines and one noncancerous primary cell type were treated with 107 small molecules comprising four different protein kinase–inhibitor scaffolds. Cellular phenotypes induced by each compound were quantified by multivariate statistical analysis of the morphology, staining intensity, and spatial attributes of the cellular nuclei, microtubules, and Golgi compartments. Principal component analysis was used to identify inhibitors of cellular components not targeted by known protein kinase inhibitors. Here we focus on a hydroxyl-substituted analog (hydroxy-PP) of the known Src-family kinase inhibitor PP2 because it induced cell-specific morphological features distinct from all known kinase inhibitors in the collection. We used affinity purification to identify a target of hydroxy-PP, carbonyl reductase 1 (CBR1), a short-chain dehydrogenase-reductase. We solved the X-ray crystal structure of the CBR1/hydroxy-PP complex to 1.24 Å resolution. Structure-based design of more potent and selective CBR1 inhibitors provided probes for analyzing the biological function of CBR1 in A549 cells. These studies revealed a previously unknown function for CBR1 in serum-withdrawal-induced apoptosis. Further studies indicate CBR1 inhibitors may enhance the effectiveness of anticancer anthracyclines. Morphology-based screening of diverse cancer cell types has provided a method for discovering potent new small-molecule probes for cell biological studies and anticancer drug candidates.

## Abstract 2

An imaging-based screen, followed by structural and functional analysis, led to the discovery of new inhibitors of carbonyl reductase 1, a potential anticancer target

## Introduction

Many current drugs were originally discovered through observation of unexpected biological activities (e.g., penicillin, benzodiazepines, sildenafil [Viagra]). Broad screens for biological function have the advantage of identifying the best “lock” for each new “key” produced by chemical variation. In contrast, the search for drug-like hits by high-throughput approaches is dominated by in vitro single-enzyme activity–based screens and single-readout cell-based assays. These approaches measure very limited regions of biological space and do not reveal potent effects on pathways not being measured directly. In order to systematize the understanding of the full activity of new small molecules, we quantified dose-dependent morphological changes induced in five cell types, thereby identifying “hit” compounds with unique activities.

The assay is based on the principle that many cellular targets are involved in the control of cellular morphology, DNA content and location, and morphology of the Golgi apparatus ([1,2,3]; C. L. Adams, D. A. Coleman, G. Cong, A. M. Crompton, K. A. Elias, et al., unpublished data). Cell-type-specific components are known to utilize distinct pathways and cellular programs to control fundamental processes affecting the features of the organelles and the overall cellular morphology. Five cell types (lung adenocarcinoma, ovarian cancer, a neuronal glioma, a prostate cancer, and endothelial cells) were included in the morphological screen. The approach has been validated by analysis of known pharmacologically active compounds from ten different mechanism of action classes (actin inhibitors, calmodulin antagonists, endoplasmic reticulum Ca^2+^ ATPase inhibitors, geranylgeranyl transferase-1 inhibitors, G-protein-coupled receptor activators, protein kinase C activators, topoisomerase II inhibitors, tubulin destabilizers, tubulin stabilizers, and kinase inhibitors). In every case, a high percentage of the compounds were accurately classified into the ten different mechanism of action groups using the Cytometrix (TM) system (C. L. Adams, D. A. Coleman, G. Cong, A. M. Crompton, K. A. Elias, et al., unpublished data).

A screen of 107 small molecules comprising four different chemical scaffolds known to inhibit protein kinases with varying selectivity and potency were selected for the Cytometrix screen. In this report, we focus on a hydroxyl-substituted analog, 3-(1-*tert*-butyl-4-amino-1H-pyrazolo[3,4-d]pyrimidin-3-yl)phenol (hydroxy-PP), of the known Src-family protein kinase inhibitor 1-*tert*-butyl-3-(4-chlorophenyl)-1H-pyrazolo[3,4-d]pyrimidin-4-amine (PP2), because it exhibited a cell response profile distinct from the known kinase inhibitors including the closely related compound PP2. Although the compound collection was dominated by kinase-inhibitor scaffolds, we identified a nonkinase target of hydroxy-PP, carbonyl reductase 1 (CBR1), an NADPH-dependent reductase. Hydroxy-PP and analogs chosen by structure-based design were used to search both for signaling pathways in which CBR1 may be involved and for potential therapeutic uses of CBR1 inhibitors.

## Results

### Selection of Chemical Library

**Figure 1:**
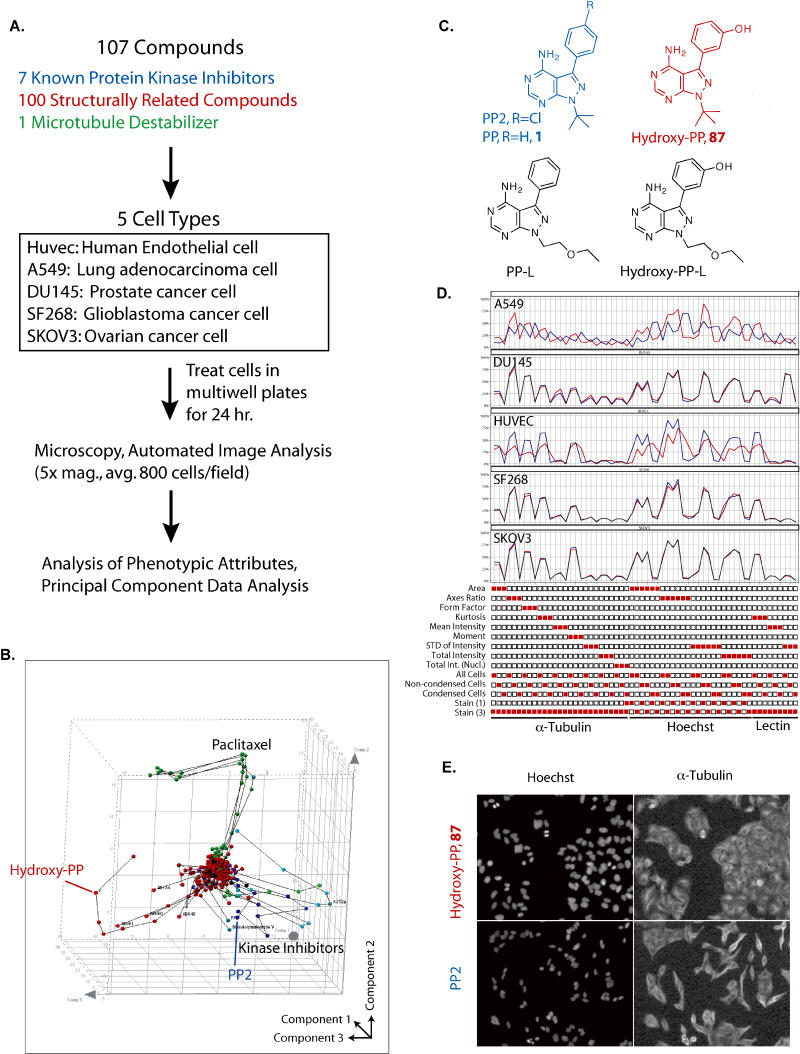
Cell Morphology–Based Screen for Biologically Active Small Molecules (A) Steps in the drug-screening process. Five human cell types, including one primary and four cancer cell lines, were treated for 24 h with the screening library that included compounds of known function and related analogs. The Cytometrix (TM) data analysis package was used to analyze microscopy data for each treatment condition. (B) PCA plot of the phenotypic attributes. Colored spheres represent a single compound at one concentration (ranging from 6 nM to 40 μM by 3-fold increases); lines connecting the spheres indicate a single compound's effects over a range of concentrations. Spheres are colored as follows: known protein kinase inhibitors (blue), paclitaxil (green), and novel compounds structurally related to the protein kinase inhibitors (red). The PCA provides aggregate variables termed “components” made up of multiple independent variables, each with a “loading factor.” These values are provided in Table S2. Structures of each compound are given in Figure S1. (C) Structures of the known kinase inhibitors PP and PP2 (blue), as well as the novel “hit compound” hydroxy-PP (red), are shown. Linker analogs of PP (PP-L) and hydroxy-PP (hydroxy-PP-L) that were used to ascertain the functional tolerance of replacing the *t*-butyl substituent at N-1 of the “hit compound” hydroxy-PP are shown. (D) Morphological attribute tabulation for cells treated with 129.4 μM PP2 (blue lines) or 0.4 μM hydroxy-PP (red lines) in each of five cell types. Data for the x-axis is grouped by the probe used (α-tubulin antibody, Hoechst dye, and lectin stain). Each of 14 attributes contributing to the magnitude of the response (y-axis) is shown as a red-filled square. (E) Visual morphology of A549 cells treated with hydroxy-PP or PP2. Hoechst dye or α-tubulin antibody was used to stain cells. The PP2-treated cells are more elongated and have more a condensed nuclear structure as compared with hydroxy-PP-treated cells.

A collection of 107 compounds containing known protein kinase inhibitors and close structural analogs were screened in the Cytometrix assay for unique phenotypic profiles suggestive of potent inhibition of cellular targets not affected by known protein kinase inhibitors (Figure 1A). The well-characterized protein kinase inhibitors (K252a [4], SKB203580 [5], VK-1911 [6], and PP2 [7]) served as positive controls and “landmarks” for the phenotypes likely to be induced by the less-characterized compounds in the collection (Figure S1 contains a complete list of all compounds tested). An advantage of screening compounds closely related to each other is the availability of a wealth of structure–activity relationships in the initial screen that provide a guide to follow-up studies aimed at improving affinity and target selectivity in a second round of chemical synthesis.

### Phenotypic Profiling Using Microscopy and Automated Image Analysis

Cellular and organelle morphology changes were measured from segmented images of cells stained with DNA and microtubule markers using algorithms that identify cell and nuclear boundaries (C. L. Adams, D. A. Coleman, G. Cong, A. M. Crompton, K. A. Elias, et al., unpublished data). Combining segmentation and intensity distribution algorithms allows acquisition of multiple shape-, texture-, and intensity-related features for each image collected. For each object identified by the segmentation algorithms, collected attributes include object location, area, perimeter, and axis ratio, as well as pixel-intensity sum, mean, variance, and kurtosis (the degree of peakedness of a distribution).

Cells undergo major morphological changes in the course of cell-cycle progression [8]. To separate these changes from ones induced by compound treatment, algorithms were used to classify cells by their cell-cycle status based on the DNA content, morphology, and condensation status. The multiple attributes of individual cells are summarized by a set of statistics that describe distributions of these attributes in a population of cells. These statistics are termed “phenotypic attributes.” The attributes used to characterize the screening compounds are listed in Table S1. These attributes were chosen for their biological information content and their low correlation with each other (C. L. Adams, D. A. Coleman, G. Cong, A. M. Crompton, K. A. Elias, et al., unpublished data).

### Phenotypic Landmarks: Compounds with Known Mechanisms of Action

Principle component analysis (PCA) was used to reduce the dimensionality in the dataset to allow visual investigation of patterns in the multivariate signature. This transformation converts a number of correlated variables into a smaller number of uncorrelated variables, or principal components, in such a way that the first few components account for as much of the variability in the dataset as possible [9]. Importantly, component values are not physical constants but are dependent on the relative “spread” of the attributes derived from all the images in the dataset.

The plot of principle components 1, 2, and 3 derived from image analysis of the effects of 107 compounds (at eight different concentrations ranging from 6 nM to 40 μM) on five cell types is shown in a scatter plot (Figure 1B). In this analysis the morphological features making up the individual components are given in Table S2. We observed four distinct “phenotypes” induced by various members of the compound collection. At the lowest concentrations tested, compounds cluster with the dimethyl sulfoxide (DMSO) and untreated controls. This region, approximately in the center of the PCA plot, constitutes the attribute profile characteristic of no effect on cellular morphology.

The remaining three phenotypic categories are characterized by compounds that exhibit distinct “trajectories” such that at increasing concentrations different attributes are enhanced, indicative of a measurable dose-dependent phenotype. The cells treated with the microtubule-polymerizing natural product paclitaxel exhibit a pronounced and highly reproducible trajectory and constitute the second phenotype observed. Each of the five cell types exhibited reproducible changes in attributes associated with tubulin staining and cell-cycle status, as expected for a microtubule-polymerizing agent [10].

A third characteristic phenotype is exemplified by the potent general kinase inhibitor K252a. The bisindolocarbazole K252a is a potent inhibitor of over 50 known kinases from diverse families [4]. This compound induced dose-dependent morphological changes in A549, DU145, HUVEC, and SF268 cells but not SKOV3 cells (data not shown). We attribute the majority of the observed changes in morphological attributes to the kinase-inhibitory activity of K252a, rather than to any off-target effects, because K252a analogs (compounds 103–107 in Figure S1) that were lacking the ability to inhibit protein kinases [11] clustered with the “no phenotype” controls. That the K252a-induced phenotype was caused by inhibition of cellular kinases is consistent with the clustering of the K252a trajectory with trajectories of other known kinase inhibitors (PP2 and SKB203580). Although the kinase targets of each of these compounds are not fully characterized, the fact that the compounds share a similar profile in the Cytometrix assay suggests that they have overlapping targets (i.e., PP2 and SKB203580 inhibit p38), as has been reported elsewhere [12].

The fourth phenotype was produced by a structurally related pyrazolopyrimidine in the collection, hydroxy-PP (compound 87 in Figure S1) (Figure 1C). The cellular attributes characteristic of this compound are distinct from the phenotypes induced by kinase inhibitors and the microtubule depolymerization inhibitors (Figure 1B). In order to best distinguish the cellular attributes unique to hydroxy-PP, a close structural analog, PP2, was used as a reference. Hydroxy-PP bears a *meta*-OH substituent, whereas PP2 has a *para*-Cl substituent on the C-3 phenyl ring [7]. Other close analogs of PP2 (Figure 1C), such as 1-*tert*-butyl-3-phenyl-1H-pyrazolo[3,4-d]pyrimidin-4-amine (PP; compound 1 in Figure S1), that lack any substituent on the C-3 phenyl ring showed a phenotypic profile comparable to PP2. (For this reason we used PP2 and PP interchangeably.) These structure–activity relationships suggested that the *meta*-OH substituent was critical for interaction with a protein target that is not affected by PP2.

The cellular effects induced by hydroxy-PP and PP2 were further examined by analysis of the quantitative attribute changes at approximately the EC_50_ value for each compound (as judged by distance in PCA space from the DMSO controls; Figure 1B). Figure 1D shows that the two compounds hydroxy-PP and PP2 exhibit indistinguishable effects on DU145, SF268, and SKOV3 cells. However, the two compounds exhibit cellular activities that are distinct from each other in A549 lung adenocarcinoma and HUVEC cells. Examples of the cell images that were analyzed by the Cytometrix algorithms and are directly relevant to the morphological differences induced by PP2 or hydroxy-PP are shown in Figure 1E. The PP2-treated A549 cells appear more elongated and slightly more condensed than the hydroxy-PP2-treated cells, leading to the quantitative differences plotted in Figure 1D. That only two of five cell types exhibited differential responses to two closely related molecules highlights the importance of including cells representing a diversity of tissue sources and genetic makeup in order to explore a wide range of possible small-molecule targets (C. L. Adams, D. A. Coleman, G. Cong, A. M. Crompton, K. A. Elias, et al., unpublished data). Attempts to directly assign the molecular target or targets responsible for the hydroxy-PP-induced changes to morphological features using compounds previously profiled using the Cytometrix™ system were not possible because the subtle differences observed were not strongly characteristic of any compounds previously profiled (unpublished data).

### Hydroxy-PP Molecular Target Identification

With no cell pathway–specific information about the target or targets of hydroxy-PP, we relied on the differential sensitivity of A549 cells to hydroxy-PP and the kinase inhibitors PP2 and PP. Our hypothesis was that hydroxy-PP and PP share targets in common based on the similarity of their Cytometrix profiles in DU145, SF268, and SKOV3 cells (Figure 1D). A corollary of this hypothesis is that hydroxy-PP targets one or more proteins in A549 and HUVEC cells that are not targeted by PP, thus leading to differential morphological attributes in A549 and HUVEC cells (Figure 1D).

As a test of the first hypothesis, we focused on identification of enzymes that are potently inhibited (IC_50_ < 1 μM) by PP and hydroxy-PP. Based on the known protein kinase–inhibitory properties of PP2 and its structural homologs, including PP [7,13], we predicted that hydroxy-PP was also a protein kinase inhibitor. We tested hydroxy-PP against four divergent protein kinase targets: the tyrosine kinase Fyn, [7,13], p38-α, protein kinase A, and protein kinase B (Table S3). The additional -OH moiety of hydroxy-PP, did not diminish kinase-inhibitory activity toward Fyn, the best known target of PP2; instead, it enhanced it such that hydroxy-PP exhibited a 5-nM IC_50_ for Fyn inhibition. Similar to what occurred with PP, hydroxy-PP did not exhibit potent inhibition of any of the three other kinases tested (Table S3).

To test the corollary hypothesis, we opted for a direct approach utilizing the differential sensitivity of A549 cells, rather than attempting to identify the unique targets of hydroxy-PP among the protein kinases, for which the differential effects are likely to be small due to the highly conserved ATP-binding pockets of protein kinases. Very few biochemical or genetic methods are available for identification of the molecular targets of small molecules in cells [14]. The most commonly used method is affinity purification by immobilization of the small molecule on a solid phase matrix. This technique requires both a high-affinity small molecule that allows stringent washing of weakly bound targets and a relatively abundant target to allow for mass spectrometry (MS)–based sequence identification. These two properties are not often found in early “hits” from random screening efforts like ours. Nonetheless, we decided to attempt affinity purification of the targets of hydroxy-PP using hydroxy-PP beads with PP beads as a negative control.

**Figure 2:**
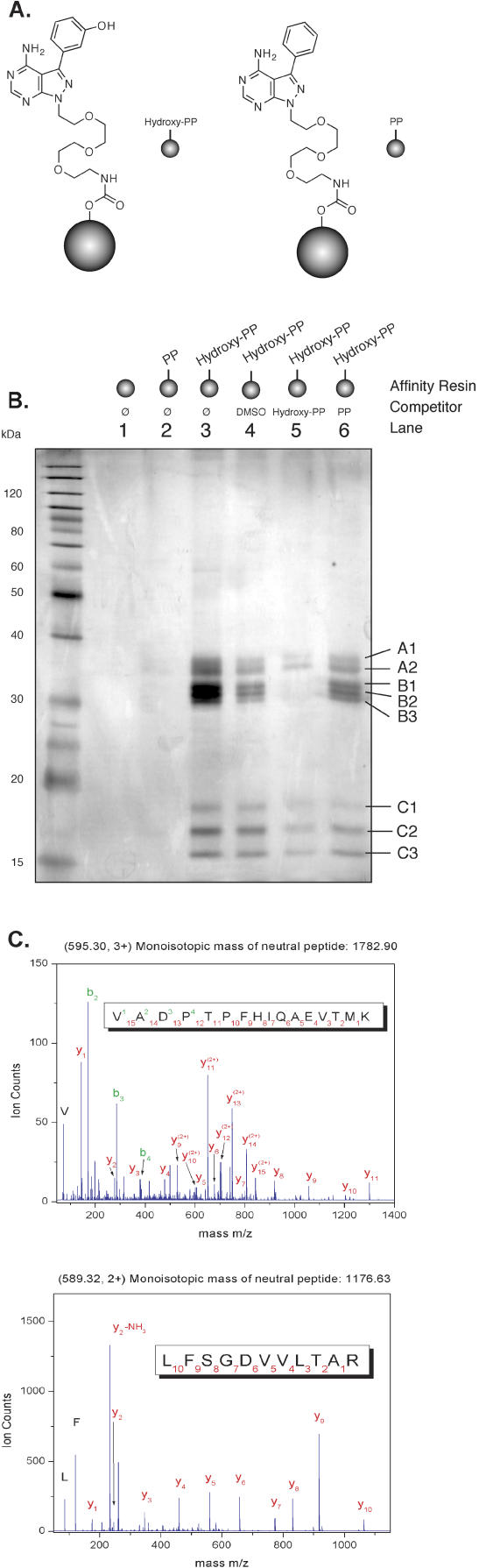
Affinity Purification and Identification of Human CBR1 (A) Reactigel beads appended with hydroxy-PP or PP (control) were used for affinity purification of hydroxy-PP protein targets. (B) Hydroxy-PP-binding proteins in A549 cell lysates. Cytosolic fractions of A549 cell lysate (1.7 mg protein each) were incubated with the indicated affinity resin, and bound proteins were resolved by SDS-PAGE (12% acrylamide gel) followed by silver staining. Untreated beads and PP-control resin samples (lanes 1 and 2) indicate little nonspecific binding. Lanes 3, 4, 5, and 6 were loaded using the hydroxy-PP resin incubated with cell lysate and the indicated competitor. Vehicle or competitor compounds (200 μM) were added to the lysate 30 min before incubation with beads (lanes 4–6). Protein of bands B1–B3 did not bind hydroxy-PP beads when pretreated with hydroxy-PP (lane 5). (C) MS/MS peptide sequencing. Two tryptic peptides from bands B1–B3 were used to identify human CBR1.

To ensure that attachment of a linker to hydroxy-PP did not destroy the target-binding properties, we synthesized a linker-containing analog of hydroxy-PP, hydroxy-PP-L, and a similar linker analog of PP, PP-L (Figure 1C). These N-1 analogs of hydroxy-PP and PP were profiled using the Cytometrix system and were found to have the same trajectories as their parent compounds, albeit with approximately 5-fold lower potencies (data not shown). This modest loss in potency is not uncommon for linker-containing analogs [16], and importantly demonstrates that the *tert*-butyl substituent at N-1 is not required for target binding. Reactigel beads presenting either hydroxy-PP or PP2 were subsequently synthesized (Figure 2A).

A wide range of buffer conditions were explored to identify conditions under which proteins specifically bound to hydroxy-PP beads but not PP2 beads or underivatized beads (Figure 2B, lane 3 versus lanes 1 and 2). Eight silver-stained protein bands at molecular weights of 15–38 kDa were retained on hydroxy-PP beads under these buffer conditions. To further distinguish those proteins that were specifically targeted by hydroxy-PP, and not by features of the linker or beads, the lysate was pretreated with DMSO, PP, or hydroxy-PP, the latter two compounds at 200 μM. Three bands (B1–B3) were not capable of binding to the hydroxy-PP beads with hydroxy-PP treatment, suggesting that these proteins were the targets of hydroxy-PP. Using matrix-assisted laser desorption/ionization (MALDI) and electrospray ionization (ESI) MS/MS sequencing, B1–B3 were each identified as human CBR1, a member of the short-chain dehydrogenase/reductase family of NAD(P)(H) oxidoreductases (Figure 2C). The presence of three forms of CBR1 with differing electrophoretic mobilities has been previously observed and is believed to result from autocatalytic modification of a lysine residue [16]. Four of the other five protein bands that were not competed off by hydroxy-PP, but that also bound to the hydroxy-PP affinity matrix, were identified as nucleoside diphosphate kinase, nucleoside diphosphate kinase 2 (nm23) and pyridoxal kinase (Table S4). Because these proteins were not eluted from the affinity beads following hydroxy-PP treatment (Figure 2B, lane 5), we concluded they were recognizing a feature of the linker used to attach hydroxy-PP to the beads and so were not pursued.

**Figure 3:**
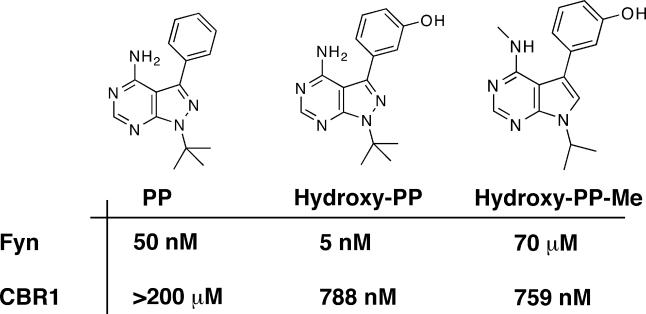
IC_50_ Values against CBR1 and Fyn Kinase Are Tabulated for PP Derivatives

To initially validate that the oxidoreductase CBR1 was indeed inhibited by hydroxy-PP, we measured CBR1 catalytic activity in vitro in the presence of hydroxy-PP or PP (Figure 3). Hydroxy-PP exhibited potent (IC_50_ = 788 nM) inhibition of CBR1-catalyzed NADPH-dependent reduction of menadione to menadiol [17]. In contrast, PP exhibited no inhibition of CBR1 activity up to its solubility limit of 200 μM. This differential inhibition of CBR1 by hydroxy-PP but not PP validated our initial hypothesis that these two compounds possess different targets.

To determine how the inhibition of CBR1 by hydroxy-PP was related to the original Cytometrix screen, the entire screening collection was re-screened in vitro for inhibition of CBR1. The only member of the collection that showed inhibition of CBR1 below an IC_50_ value of 1 μM was hydroxy-PP (data not shown). These screening data suggest that the morphology-based screen provided an efficient measure of the inhibitory potential of CBR1 inhibition, even though the screen included no direct measurements designed to read out CBR1 function. To determine whether the absence of an observable differential effect of hydroxy-PP, as compared with PP2, on SKVO3, DU145, and SF268 cells was due to an absence of CBR1 expression in these cells, we carried out a protein immunoblot analysis of CBR1 expression levels in each of the cell types analyzed by Cytometrix. Each of the cell types expressed CBR1 at approximately equal levels (Figure S2), suggesting that multiple factors other than expression level regulate CBR1 activity.

### Structural Characterization of Hydroxy-PP–CBR1 Complex

In order to develop a pharmacological agent that specifically inhibits CBR1, we addressed the target specificity of hydroxy-PP. In particular, hydroxy-PP's ability to potently inhibit the cytoplasmic tyrosine kinase Fyn (IC_50_ = 5 nM) in addition to CBR1 (IC_50_ = 788 nM) makes it a poor tool for probing CBR1 functions exclusively. We overexpressed human CBR1 in Escherichia coli and attempted crystallization of the protein in the presence of hydroxy-PP in an effort to enhance design of a selective CBR1 inhibitor. Within 2 d at room temperature, good diffracting crystals of the orthorhombic space group P2_1_2_1_2_1_ were obtained by vapor diffusion from 100 mM sodium-2-(N-ethylmorpholino)ethanesulfonate (pH 6.5), 2.0 M ammonium sulfate, and 5% PEG 400. Orthorhombic crystals of CBR1–hydroxy-PP diffracted to 1.1 Å. The structure was solved by molecular replacement with the AMoRe program [18] using a modified porcine carbonyl reductase [19] model and refined with SHELXL [20] to 1.24 Å with a crystallographic R-factor of 10.3% and a free R-factor of 13.4%.

Human CBR1 shows very high structural similarity to porcine carbonyl reductase, whose sequence is 85% identical to human CBR1 [21]. Although NADP(H) was not present during purification of the enzyme from E. coli nor added to the crystallization experiments, one molecule of NADP was found to be bound in the CBR1–hydroxy-PP structure. The same occurrence has been reported for the structure of porcine carbonyl reductase [19]. Hydroxy-PP binds to the substrate-binding site of CBR1, with the pyrazolopyrimidine core of hydroxy-PP mainly surrounded by hydrophobic residues (Trp229, Met141, and Ile140). The phenolic hydroxyl group of hydroxy-PP, however, points deep into the substrate-binding pocket and interacts with Tyr139 and Ser193 of the catalytic triad. The phenolic oxygen is positioned 2.5 Å from O^η^ of Tyr139 and 2.5 Å from O^γ^ of Ser193, thus indicating strong hydrogen bonding. The C4 carbon of the NADP(H) nicotinamide ring is positioned 3.2 Å from the *meta*-hydroxy carbon. This four-point geometry is iso-structural to the structures of other short-chain dehydrogenase substrate complexes (e.g., PDB 2AE2, 1FDS, and 1HZJ) and suggests a substrate-like binding mode of hydroxy-PP. Importantly, the structure of CBR1–hydroxy-PP provides a molecular understanding of the basis for the strong dependence of CBR1 inhibition on the presence of a hydroxyl moiety, as this functional group serves as a key interaction determinant with the catalytic machinery of CBR1. The binding mode of hydroxy-PP in CBR1 also explains the tolerance of the pyrazolopyrimidine core to derivatization at N-1, which allowed attachment of hydroxy-PP to solid support and affinity purification of CBR1.

### Design and Synthesis of a CBR1-Selective Inhibitor

**Figure 4:**
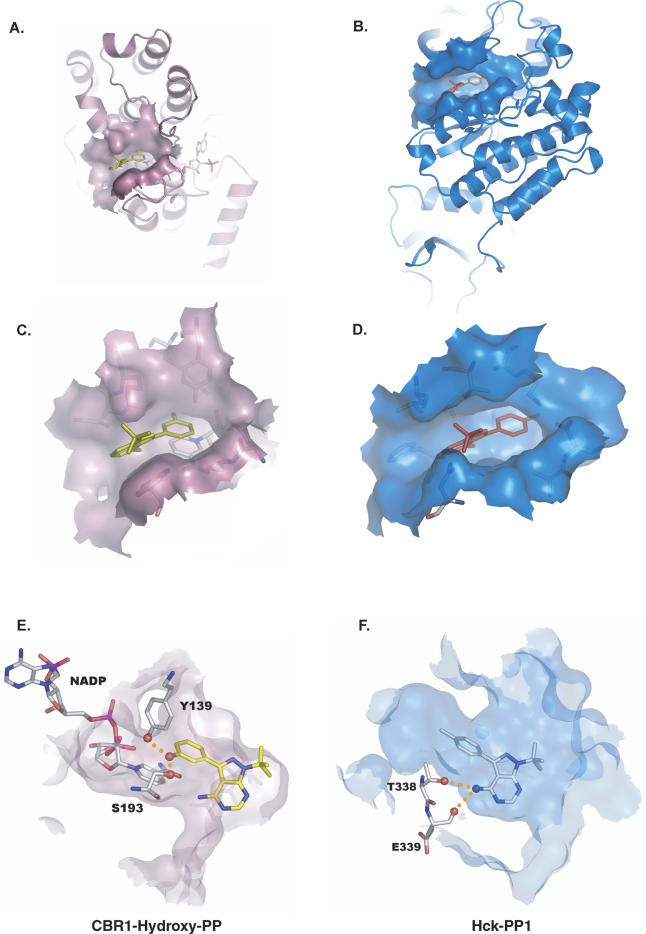
Co-Crystal Structures of CBR1–Hydroxy-PP and Hck–PP1 (A, C, and E) show the binding mode of hydroxy-PP in co-crystals with CBR1. The inhibitor is oriented with its *t*-butyl group partially exposed to solvent and points toward the surface of the protein. The phenolic moiety of the inhibitor binds deeply within the substrate-binding pocket and makes close contacts to Ser193 and Tyr139 of the catalytic triad and the bound cofactor NADP. (B, D, and F) show the binding mode of the kinase inhibitor PP1 in complex with Hck. PP1 occupies the ATP-binding pocket as an adenosine analog. Although both protein structures show different folds (A and B), the morphology of CBR1- and Hck-binding sites are similar, and inhibitors hydroxy-PP and PP1 bind to these sites with similar shape complementarity (C and D). Key H-bond interactions between hydroxy-PP and the Ser193 and Tyr139 of CBR1 are indicated (E). The exocyclic amine of PP1 in complex with Hck makes essential H-bonds with the main-chain carbonyl oxygen of Glu339 and the side-chain oxygen of Thr338 (F). Disruption of this key H-bonding interaction by derivatization of the exocyclic amine destroys kinase affinity. The figure was prepared using the PyMol 2002 graphics system (DeLano Scientific, San Carlos, California, United States).

The availability of the X-ray structures of hydroxy-PP bound to CBR1 and of 1-*tert*-butyl-3-p-tolyl-1H-pyrazolo[3,4-d]pyrimidin-4-amine (PP1) bound to the tyrosine kinase Hck [22] afforded us the opportunity to compare how two virtually identical small molecules (PP1 and hydroxy-PP) are able to bind to two completely structurally and functionally unrelated enzyme targets (Figure 4A and 4C versus 4B and 4D). Strikingly, the two co-crystal structures show grossly isosteric active site surfaces that are complementary to the two pyrazolopyrimidines. The geometries of both the pyrazolopyrimidine C-3 phenyl bond and orientation within the binding clefts are conserved within the two complexes. This finding complicates the design of a hydroxy-PP analog that cannot bind to protein kinases because the two binding clefts are highly similar in geometry. To design an analog of hydroxy-PP containing substituents that would disrupt protein kinase binding, we focused on electronic rather than steric aspects of the complexes. The exocyclic amine of PP2 is known to make key H-bond interactions with O^γ^ of Thr338 and O of Glu339 in the linker region of the protein kinase active site pocket (Figure 4F) [22]. We designed and synthesized a mono-methyl-substituted version of hydroxy-PP, 3-(7-isopropyl-4-(methylamino)-7H-pyrrolo[2,3-d]pyrimidin-5yl)phenol (hydroxy-PP-Me), predicted to disrupt this key H-bonding interaction in kinases. Importantly, structural analysis of the hydroxy-PP–CBR1 co-crystal structure revealed a small space in the active site capable of tolerating a methyl substituent at this position (Figure 4E).

Indeed, hydroxy-PP-Me maintained potent inhibition activity against CBR1 (IC_50_ = 759 nM) but was an extremely poor inhibitor of the cytoplasmic tyrosine kinase Fyn (IC_50_ > 70 μM; see Figure 3). Because hydroxy-PP-Me lacks an H-bond donor group known to be key for potent protein kinase inhibition, we anticipate that it is an extremely poor inhibitor of all cellular protein kinases. A small screen against four protein kinases (Fyn, p38-α, protein kinase A, and protein kinase B) (see Table S3) has shown that none are inhibited by hydroxy-PP-Me. Further in vitro screens against a large panel of protein kinases will be required to experimentally confirm this assertion.

### CBR1 and Cancer Therapy

The carbonyl reductase CBR1 was first isolated from brain [17] and has been associated with two cellular functions: (1) detoxification of xenobiotics, such as the anthracycline daunorubicin and (2) metabolism of ketone-containing cellular messengers, such as prostaglandin E (reviewed in [23]). Genetic studies that have intended to uncover the in vivo function of this enzyme have focused on the xenobiotic detoxification activity of the enzyme. CBR1 converts daunorubicin into daunorubicinol, a compound that lacks the anti-proliferative activity of the parent daunorubicin and is cardiotoxic. Thus, metabolism of daunorubicin by CBR1 is thought to be responsible for the severe cardiotoxicity associated with daunorubicin treatment. In support of this function, mice heterozygous for a null allele of CBR1 show reduced sensitivity to anthracycline-induced cardiotoxicity because reduced CBR1 expression produces lower levels of doxorubicinol (CBR1 homozygous null mice are nonviable) [24]. Further support for this model comes from transgenic mice overexpressing CBR1, which exhibit increased cardiotoxicity associated with doxorubicin treatment [23]. It has been suggested [23] that because CBR1-dependent metabolism of the anthracyclines doxorubicin and daunorubicin reduces their efficacy in tumor-cell killing, a pharmacological inhibitor of CBR1 should potentiate anthracycline-induced cancer-cell killing.

**Figure 5:**
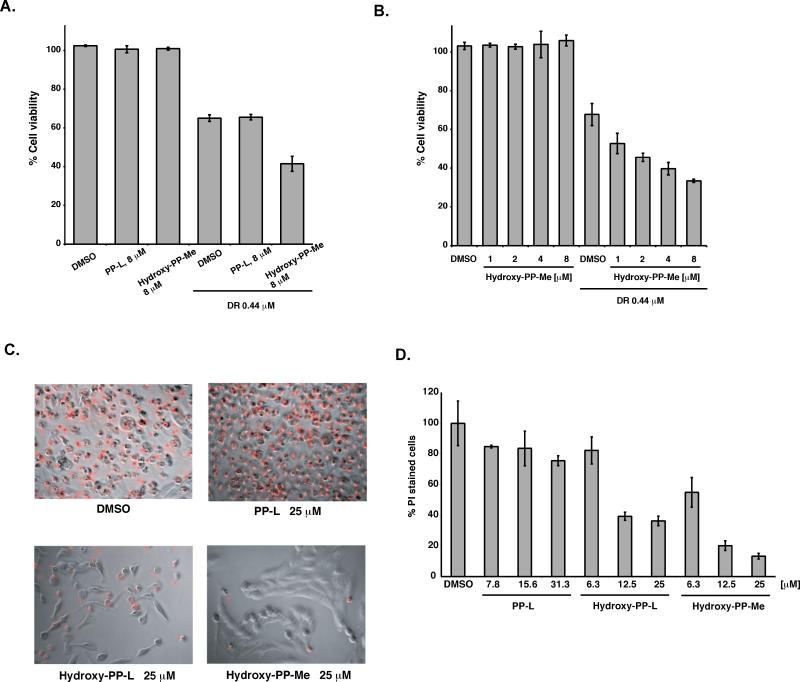
CBR1 Inhibitors Enhance Daunorubicin-Mediated A549-Cell Killing, yet Prevent Apoptosis in Serum-Starved Cells (A) Cell viability as a function of drug treatment. DMSO, PP-L (8 μM), and hydroxy-PP-Me (8 μM) do not have a pronounced effect on cell viability when used alone. Daunorubicin (DR) alone induces a moderate decrease in cell viability that is accentuated by concomitant treatment with hydroxy-PP-Me. (B) Cell viability decreases dose dependently with concomitant daunorubicin (DR) treatment. Daunorubicin treatment induces a moderate decrease in cell viability when used alone. Hydroxy-PP-Me (1–8 μM) induces a dose-dependent decrease in cell viability with concomitant daunorubicin treatment. (C) PI staining of dead cells is appreciably decreased in serum-starved cells treated with CBR1 inhibitors. A high number of cells were PI stained 65 h following serum starvation in both control and PP-L treated conditions (top). Cells treated with the CBR1 inhibitors hydroxy-PP-L or hydroxy-PP-Me during serum starvation show appreciably less staining (bottom). (D) Quantification of PI-stained cells by fluorescence measurement 65 h following serum starvation. Hydroxy-PP-L and hydroxy-PP-Me induce a dose-dependent decrease in PI staining; whereas PP-L does not.

To test this hypothesis, we measured the ability of hydroxy-PP-Me and PP-L to block CBR1-mediated metabolism of daunorubicin in A549 cells using cell killing as a measure of the cellular status of daunorubicin metabolism. The potent kinase inhibitor PP-L, which does not inhibit CBR1, was used as a negative control to measure general toxicity of combining daunorubicin with a structurally related but inactive molecule. A549 cells were treated at a daunorubicin concentration (440 nM) corresponding to an approximate IC_50_ for cell killing as a single agent such that enhanced cell killing could be scored. Concentrations of PP-L and hydroxy-PP-Me that exhibited minimal cytotoxicity on A549 cells (8 μM) when used alone were selected for combination with daunorubicin. Figure 5A shows cell viability results measured by an alamarBlue reduction assay for A549 cells following drug treatments. Hydroxy-PP-Me induced a 25% enhancement of daunorubicin-mediated A549-cell killing consistent with its ability to inhibit CBR1-mediated daunorubicin metabolism. In contrast, PP-L exhibited no enhancement of cell killing, further suggesting the need for CBR1 inhibition to enhance daunorubicin-mediated cell killing. Although the observed 25% enhancement of daunorubicin-mediated cell killing is modest, the hydroxy-PP-Me dose dependence (Figure 5B) of this effect is further evidence that it is CBR1 mediated rather than a general toxic response.

### Anti-Apoptotic Effect of Hydroxy-PP-Me Links CBR1 Activity to Serum-Withdrawal-Induced Cell Stress

The embryonic lethal phenotype of CBR1^–/–^ mice suggests that the enzyme plays a nonredundant role in cell signaling during embryogenesis and development. In order to search for unknown biological roles for CBR1, we utilized the two structurally related pyrimidine-based inhibitors of CBR1 hydroxy-PP-L and hydroxy-PP-Me. The former inhibits CBR1 and protein kinases, whereas the latter lacks the kinase-inhibitory action while maintaining CBR1-inhibitory activity. PP-L was included as a negative control. Thus, the three compounds together constitute a probe set for assessment of CBR1 involvement in a variety of cellular processes.

We chose to focus on signals that induce apoptosis in A549 adenocarcinoma cells because this endpoint is important for many cell-fate decisions, including those relevant to cancer and inflammation [25]. CBR1 has been directly implicated in redox reactions leading to H_2_O_2_ generation, a known stimulus for apoptosis [26]. A549 cells were subjected to a wide range of cell stresses previously shown to induce apoptosis: (1) interferon-γ + Fas ligand, (2) H_2_O_2_, (3) interleukin-1β, (4) serum withdrawal, or (5) interleukin-1β + serum withdrawal. The CBR1 inhibitors hydroxy-PP and hydroxy-PP-Me showed no enhancement of induction of apoptosis by any of these conditions (data not shown). However, both inhibitors were able to block A549-cell apoptosis induced by serum withdrawal [27]. Propidium iodide (PI) staining and phase contrast images show that A549 cells 65 h following serum withdrawal undergo virtually 100% apoptosis whereas cells treated with hydroxy-PP-Me are almost completely protected against apoptosis, as judged by the number of PI-negative cells (Figure 5C). The dual CBR1–protein kinase inhibitor hydroxy-PP-L also protects A549 cells against serum-withdrawal-induced apoptosis, although to a lesser extent than hydroxy-PP-Me.

To confirm that inhibition of CBR1 by hydroxy-PP-Me was responsible for the anti-apoptotic effects in serum-starved A549 cells, we turned to RNA interference (RNAi) as a means to validate the role of CBR1 in the process. Three types of 21-nt RNAi that target human CBR1 were tested for their ability to knock down CBR1 expression, and their effectiveness was confirmed with an anti-CBR1 antiserum Western blot (Figure S3A). The anti-apoptotic effects of these RNAi elements that target CBR1, as opposed to control RNAi elements that target an irrelevant target found in A549 cells, further validate that hydroxy-PP-Me is a potent CBR1 inhibitor in cells and that CBR1 is involved in serum-withdrawal-induced apoptosis (Figure S3B).

To begin to determine whether the observed serum-withdrawal-induced apoptosis in A549 cells is mediated by the well-characterized p53 pathway, we used RNAi elements targeting p53 and showed that loss of p53 also protects A549 cells from serum-starvation-induced apoptosis (Figure S3B). A connection between p53-mediated cell death and another NADH-dependent reductase (NQO1) has been discovered recently [28]. This reductase is a p53-binding partner, and, upon reactive oxygen species generation in cells, p53 is released from NQO1, which then induces apoptosis. We sought to determine whether CBR1, another nicotinamide-dependent reductase, could act similarly as part of a p53 complex by measuring p53 levels following serum-withdrawal-induced apoptosis. These studies revealed no ubiquitination nor a decrease in p53 concentration following CBR1 inhibition as when NQO1 inhibitors are added to cells, suggesting that CBR1's involvement with the apoptotic machinery does not follow the pattern established for other oxidoreductases (data not shown).

## Discussion

We have explored a conceptually new approach for the discovery of novel potent and selective inhibitors of cellular proteins. Rather than attempt to search extensively through chemical space using large chemical libraries, we greatly expanded the amount of biological target space sampled in a single screen with only a limited collection (107) of small drug-like molecules of limited chemical diversity. The morphology-based screen led to the identification of hydroxy-PP, which exhibited a multi-dimensional morphological signature distinct from a known kinase inhibitor of related structure (PP2). Not surprisingly, hydroxy-PP inhibited protein kinases based on its similarity in structure to the known Src-family kinase inhibitor PP2. In order to discover the new target of hydroxy-PP, an affinity-based screen was carried out. This approach revealed a new protein target for hydroxy-PP that was not inhibited by PP2. The target identified for hydroxy-PP and validated by X-ray crystallography was not a protein kinase, but the NADPH-dependent oxidoreductase, CBR1.

The surprising ability of a protein kinase inhibitor to cross-inhibit a member of a completely distinct protein family by simple hydroxyl-group substitution was rationalized based on X-ray structure analysis of the hydroxy-PP-binding site of CBR1. The protein kinase pocket occupied by PP in Hck is closely related, in terms of overall shape, to the hydroxy-PP-binding site of CBR1. Moreover, the hydroxyl group on hydroxy-PP makes contact with two catalytically essential residues in CBR1, thus providing direct structural insight into the original structure–activity relationships identified by morphology-based screening.

Genetic models have confirmed the importance of CBR1 in producing cardiac myocyte–toxic metabolites of important anthracyclines, such as daunorubicinol [29]. The discovery of a small-molecule inhibitor of CBR1 and the evidence of improved cell killing by daunorubicin in conjunction with hydroxy-PP-Me of lung adenocarcinoma cells now allows for assessment of this potential improvement to current adjuvant therapy for treating breast cancer and childhood leukemias by reducing or preventing the cardiotoxicity currently associated with anthracycline therapy. Further studies in mice treated with daunorubicin and hydroxy-PP-Me are being initiated to investigate possible cardiotoxicity reduction once the in vivo organ distribution and pharmacokinetics of hydroxy-PP-Me are determined. In particular, distribution of hydroxy-PP-Me in the heart may be essential to block daunorubicin-induced cardiotoxicity because it is thought that CBR1 activity in the heart is responsible for producing the high local concentration of daunorubicinol that is toxic to cardiomyocytes.

The discovery of a potent and selective CBR1 inhibitor also provides a powerful tool for discovery of pathways in which CBR1 plays a role. The CBR1 knockout is lethal, preventing such studies by genetic means. In a limited screen conducted so far, one process found to be most sensitive to CBR1 inhibition was serum-withdrawal-induced apoptosis. The selective CBR1 inhibitor hydroxy-PP-Me was able to block more than 90% of apoptosis induced by this stimulus. This pathway was not known to be dependent on the oxidoreductase CBR1, thus validating that the compounds discovered in such screens can lead to chemical tools capable of uncovering novel functions of key signaling regulators.

The apparent paradox that CBR1 serves a pro-apoptotic function during serum-withdrawal-induced apoptosis but has a protective function when cells are challenged with anthracyclines is a consequence of the different roles of the natural and unnatural (anthracycline) substrates of CBR1. The means by which inhibition of CBR1 causes increased cell death in anthracycline-challenged cells is through blocking the anthracycline-detoxification activity of CBR1. However, the anti-apoptotic effect of blocking CBR1 activity during serum-withdrawal-induced apoptosis is unclear. Inhibition of CBR1 may prevent generation of H_2_O_2_ via cellular quinone reduction by CBR1 and the subsequent comproportionation reaction of a cellular hydroquinone species and O_2_ to form H_2_O_2_ [30]. Further experiments to determine the cellular substrates of CBR1 that are involved in execution of serum-withdrawal-induced apoptosis using methods for directly radiolabeling reduction products are being pursued [31].

The present study suggests that current focused collections of small molecules for cell-based screens contain potent ligands for cellular targets that might be missed when screens based on single readouts or single-cell processes are used. Efforts to include more specific readouts in the microscopy-based screens, such as use of phospho-specific antibodies for reading out specific kinase activation, have been recently described [1]. Other fluorescent readouts, including tagged fusion proteins for visualization of individual protein trafficking events and fluorescent sensors of metals such as Ca^2+^and Zn^2+^, and other visualization methods may increase the information content available for predicting targets of novel small molecules.

## Materials and Methods

### 

#### Cell culture and cell plating

SKOV3 (ovarian epithelial cancer), A549 (lung epithelial cancer), and SF268 (central nervous system epithelial cancer) were chosen for their broad genetic diversity and obtained from the National Institutes of Health. These human cells were grown and maintained in RMPI medium (Mediatech, Herndon, Virginia, United States) with 5% fetal calf serum (FBS, HyClone, Logan, Utah, United States). DU145 (prostate epithelial cancer) cells ( ATTC, Manassas, Virginia, United States) were maintained in MEM with 5% FBS. HUVEC cells (VEC Technologies, Rensselaer, New York, United States) were maintained in MCDB131 medium (VEC Technologies) with 10% FBS. For the assays 1,000–1,800 trypsinized (Mediatech) cells per well (depending on cell type) were plated in 384-well plates (Corning Costar, Acton, Massachusetts, United States) using a Multidrop (Thermo Labsystems, Beverly, Massachusetts, United States) and incubated for 24 h, the approximate doubling time. Six cell plates were made for each cell line.

#### Compounds

Compound stocks were maintained in DMSO. Compound information was stored in ActivityBase (ID Business Solutions, Guildford, United Kingdom). Compounds were serially diluted in 384-well drug plates to achieve eight concentrations diluting 3× in DMSO between wells using a Multimek (Beckman Coulter, Fullerton, California, United States). Wells were reserved on every drug plate for negative and positive controls. Negative-control wells contained DMSO only and the positive-control wells received a titration of paclitaxel, a tubulin stabilizer (Sigma-Aldrich-Fluka, St. Louis, Missouri, United States). For known kinase inhibitors for which we possessed information about the IC_50_ against known targets, the starting concentration was customized such that the IC_50_ would be in the middle of the dose-response (eight 3-fold dilutions) curve. Small molecules were tested at 40 μM on cells based on the logic that cellular effects on cells at greater than 100 μM concentration are most likely nonspecific and that we would most likely get a full-dose response after eight 3-fold dilutions (6 nM to 40 μM final concentrations).

#### Compound addition

Compounds were added to cell plates from drug plates. Compound mixed with medium was added to the cells to achieve a 0.4% DMSO concentration on prepared cell plates using a PlateTrak (CCS Packard, Torrance, California, United States). Treated cells were incubated for another 24 h, the compound exposure time.

#### Staining

Cells were stained for visualization of the nuclei, Golgi apparatus, and microtubules. Cells were fixed with 4% formaldehyde (Polysciences, Warrington, Pennsylvania, United States) for 1 h; washed in TBS (Teknova, Half Moon Bay, California, United States); blocked in 0.01% Triton X-100 (ICN Biomedicals, Irvine, California, United States) and 1% BSA in TBS (Teknova) and then left to incubate for 1 h; stained with 0.01% Triton X-100, 0.1% BSA, 5 μg/ml Hoechst 33342 (Molecular Probes, Eugene, Oregon, United States), 5 μg/ml FITC-Lectin Lens Culinaris (Sigma-Aldrich-Fluka), and 3 μg/ml Rhodamine Red–labeled monoclonal antibody DM1α (courtesy of Tim Stearns) for 1 h; and then washed in TBS. All of these steps were performed with a PlateTrak using an ELx405 microplate washer (Bio-Tek Instruments, Winooski, Vermont, United States). This was done in triplicate for each cell line.

#### Imaging

Cells were imaged on an inverted Axiovert 100M epifluorescent microscope (Carl Zeiss, Oberkochen, Germany) with a 5× objective and a Xenon lamp (Sutter Instruments, Novato, California, United States). A 5× magnification was chosen (rather than 40×) primarily as a function of the need to acquire a large number of individual cells in order to gather statistically relevant data on relatively rare subpopulations. The number of cells in a randomly acquired 40× image is not sufficient to make meaningful statistical measurements across many cells. Metamorph (Universal Imaging, Downingtown, Pennsylvania, United States) was used to control the motorized x, y, z stage (Prior Scientific, Rockland, Massachusetts, United States) that moved the plate to each well, autofocused, and took three successive fluorescent images with an Orca 100 camera (Hamamatsu, Hamamatsu City, Japan). Exposure times were set to minimize the number of saturated pixels in the image. Images of the negative-control wells typically contained 800 to 1,200 cells, depending on the cell line.

#### Image analysis

Custom software was used to segment objects and extract attributes. Segmentation of nuclei used a gradient method for edge detection, the microtubules were segmented using watershed, and the Golgi apparatus was segmented by expanding the mask from the nuclei. Object attributes are listed in Table S1.

#### Object classification

Two algorithms using object attributes from the nuclei classify objects into different phases of the cell cycle. The cell-cycle algorithm classifies each cell by total intensity of its nuclei (DNA content) as Gap 1, Synthesis, or Gap 2. The condensation algorithm classifies each cell as condensed or not condensed using the mean intensity and area of the nuclei. Nuclei condensation is a surrogate marker for mitosis; meaning that condensed cells are typically mitotic cells but may be rounded-up cells.

#### Data storage and analysis

Custom software was written to automatically identify, register, organize, and analyze the images. Results from image analysis were automatically stored in an Oracle (Redwood Shores, California, United States) database. Information about the experimental conditions was manually entered into the database. Finally, software was written to compile compound information from ActivityBase, experiment design, image analysis, and data analysis into one report. SpotFire DecisionSite (Somerville, Massachusetts, United States) was used to visualize the attribute and PCA data.

#### Quality control

Quality metrics computed on the negative- and positive-control wells were used to determine the overall quality of the plate. Poor-quality plates were discarded and new plates were made. Image statistics—background, contrast, and saturation–were measured, and poor quality images were discarded. Results were aggregated over good images from replicated cell plates.

#### Chemicals and chemical synthesis

Reactigel 6X was purchased from Pierce Biotechnology (Rockford, Illinois, United States). NADPH, glutathione, menadione, and daunorubicin were purchased from Sigma. AlamarBlue was purchased from Biosource (Camarillo, California, United States) International. Boronic acids were purchased from Combi-Blocks (San Diego, California, United States), and palladium complexes were purchased from Strem Chemical (Newburyport, Massachusetts, United States). Other starting materials and synthetic reagents were purchased from Aldrich unless otherwise noted.

4-Amino-1-*tert*-butyl-3-phenylpyrazolo[3,4-*d*] pyrimidines (PP2 and hydroxy-PP) were synthesized according to Hanefeld et al.[32]. Benzyl bromide was used to protect the hydroxyl group for the hydroxy-PP synthesis. Hydrogenation (10% palladium on carbon) resulted in benzyl deprotection to yield hydroxy-PP.

Hydroxy-PP: Colorless powder; ^1^H NMR (400 MHz, DMSO-*d*6) δ 9.69 (brs), 8.22 (1H, s), 7.33 (1H, dd, *J* = 7.5, 7.5 Hz), 7.05 (2H, m), 6.86 (1H, dd, *J* = 7.5, 2 Hz), 5.74 (s), 1.73 (9H, s). ^13^C NMR (100 MHz, DMSO-*d*6) δ 158.1 (s), 157.8 (s), 154.6 (d), 153.8 (s), 141.7 (s), 134.5 (s), 130.2 (d), 118.9 (d), 115.6 (d), 115.0 (d), 98.5 (s), 59.6 (s), 28.7 (q). HR-EIMS calculated for C_15_H_17_N_5_O 283.1433 found 283.1434.

Chemical probes with a linker (PP-L and hydroxy-PP-L) were synthesized by the coupling reaction of the corresponding pyrazolo[3,4-*d*] pyrimidines with 1-bromo-11-*tert*-butoxycarbamoyl-3,6,9-trioxaundecane by treatment with sodium hydride.

Hydroxy-PP-Me was synthesized in three steps from 4-chloro-5-iodo-7H-pyrrolo[2,3-*d*]pyrimidine (compound 1 in Figure S4), with a 50% overall yield. Compound 1 was synthesized as previously described [33]. Mitsunobu alkylation of compound 1 proceeded efficiently to yield the N-alkylated pyrrolopyrimidine (compound 2 in Figure S4). S_N_aryl reaction of this product with methylamine in THF provided compound 3 (Figure S4), which was subsequently coupled to *meta*-hydroxyphenylboronic acid under Suzuki conditions to afford hydroxy-PP-Me.

To produce 4-chloro-7,7a-dihydro-5-iodo-7-isopropyl-4aH-pyrrolo[2,3-*d*]pyrimidine (compound 2 in Figure S4), the following steps were taken. To a dry 50-ml round-bottom flask was added 4-chloro-5-iodo-7H-pyrrolo[2,3-*d*]pyrimidine prepared as previously described [33] (0.5 g, 1.78 mmol) and PPh_3_ (0.84 g, 3.2 mmol). The materials were dried under high vacuum for 20 min, and the flask was purged with argon. THF (30 ml) and isopropanol (0.3 ml, 3.9 mmol) were added and the flask was cooled in an ethylene glycol/dry-ice bath, and diisopropyl azodicarboxylate (0.47 g, 2.3 mmol) was added drop by drop to the stirred solution. After 18 h, the volatiles were evaporated in vacuo and the resultant oil was dissolved in ethyl acetate (50 ml) and 50% saturated sodium bicarbonate (50 ml). The organics were extracted with ethyl acetate (3 × 50 ml), dried with sodium sulfate, and evaporated in vacuo to yield an orange oil. Silica gel chromatography (ethyl acetate:hexanes) afforded the desired product as a yellow solid (480 mg, 84% yield). ^1^H NMR (399.6 MHz, CDCl_3_) δ 1.5 (6H, d, J = 6.4 Hz), 5.1 (1H, sp, J = 6.8 Hz), 7.4 (1H, s), 8.6 (1H, s).

To produce 7,7a-Dihydro-5-iodo-7-isopropyl-N-methyl-4aH-pyrrolo[2,3-*d*]pyrimidin-4-amine (compound 3 in Figure S4), 4-Chloro-7,7a-dihydro-5-iodo-7-isopropyl-4aH-pyrrolo[2,3-d]pyrimidine (0.3 g, 0.93 mmol) from above was placed within a 15-ml pressure tube. Methylamine (2 M) in THF (15 ml) was added, and the reaction was left to stir overnight. The volatiles were removed in vacuo, and the residue was dissolved in methanol; then 5 ml of silica gel was added, and the volatiles were removed in vacuo. The adhered product was purified by silica gel chromatography (ethyl acetate:hexanes), and the requisite fractions were pooled and evaporated in vacuo to yield the desired product (0.25 g, 85% yield). ^1^H NMR (399.6 MHz, CDCl_3_) δ 1.43 (6H, d, J = 6.8 Hz), 3.13 (3H, d, J = 4.8 Hz), 5.0 (1H, sp, J = 6.8 Hz), 7.02 (1H, s), 8.35 (1H, s).

To produce hydroxy-PP-Me, 7,7a-Dihydro-5-iodo-7-isopropyl-N-methyl-4aH-pyrrolo[2,3-*d*]pyrimidin-4-amine (0.15 g, 0.475 mmol) from above was placed in a 50-ml round-bottom flask in which 12 ml of dimethoxy ethylene glycol was added. 3-Hydroxyphenylboronic acid (0.262 g, 1.9 mmol, predissolved in 3.3 ml of ethanol) was added at once and was followed by 1.9 ml of saturated aqueous sodium carbonate. Pd^0^(PPh_3_)_4_ (55 mg, 47 μmol) was added to the reaction; the vessel was purged with argon and set to stir at 80 °C for 48 h. The reaction was subsequently cooled and filtered through a bed of diatomatious earth (Celite, Sigma-Aldrich). The filtrate was evaporated in vacuo, and the residual material was adhered to silica gel using ethyl acetate as a solvent. Silica gel chromatography (ethyl acetate:hexanes) and evaporation in vacuo of the requisite fractions yielded the desired product (94.8 mg, 70.7% yield). ^1^H NMR (399.6 MHz, d^6^-DMSO) δ 1.76 (6H, d, J = 6.8 Hz), 5.03 (3H, d, J = 4.8 Hz), 5.34 (1H, sp, J = 6.4 Hz), 5.53 (1H, q, J = 4.8 Hz), 6.73 (1H, m), 6.85 (1H, m), 7.25 (1H, app t, J = 7.6 Hz), 7.37 (1H, s), 7.59 (1H, s).

#### Cell lines

A549 cells were purchased from ATCC and cultured in the F-12K medium with 10% of FBS. Cells were maintained at 37 °C in an atmosphere of 5% CO_2_. Adherant cells were released for passaging using an isotonic Trypsin solution (0.25% Trypsin, 0.02% Versene). Rabbit anti-human CBR1 antibody was obtained from Dr. Tsuchida [34]. The CBR expression vector, pET-11aCR, was obtained from Dr. Wermuth [35].

#### Preparation of cell extracts

Cells were collected and washed with PBS buffer once then sonicated in buffer A containing 50 mM Tris-HCl (pH 7.4), 2 mM dithiothreitol, 5 mM EDTA, 5 mM EGTA, 20 mM MgCl_2_, and 1 μl/ml protease inhibitor cocktail Set III (Calbiochem, San Diego, California, United States). The solution was centrifuged for 15 min at 10,000 *g* and 4 °C. The supernatant was recovered and loaded on the affinity matrices or stored at –80 °C.

#### Preparation and use of affinity reagents

Pyrazolopyrimidines were coupled to Reactigel 6X beads at a calculated final concentration of 10–50 μmol/ml of resin. They were stored at 4 °C as a 50% (v/v) slurry in ethanol. 20 μl of the suspension was washed with 1 ml of buffer A with 200 mM NaCl and 0.1% IGEPAL CA-630 (Sigma), then the cell supernatant (1–2 mg protein) was added. After 1 h incubation at 4 °C, the beads were washed with the same buffer followed by addition of 40 μl of 1× Laemmli sample buffer.

#### Electrophoresis

Proteins bound to the affinity matrix were recovered with 1× Laemmli sample buffer. Following heat denaturation for 3 min, the bound proteins were separated by 12% SDS-PAGE followed by immunoblotting analysis or silver staining. Silver staining was performed with the following parameters: fixative, 250 ml of 50% methanol; rinse, MilliQ purified water (Millipore, Billerica, Massachusetts, United States) containing 10 μM DTT, followed by 0.1% AgNO_3_ in MilliQ water (w/v); developer, 15 g of Na_2_CO_3_ in 500 ml of MilliQ water containing 250 μl of 37% formaldehyde.

#### Protein identification: Materials

Siliconized 0.65-ml tubes from PGC Scientifics (Frederick, Maryland, United States) were washed with methanol and water prior to use. Reverse-phase packing material was from Phenomenex (Torrance, California, United States); fused-silica capillary tubing was purchased from Dionex (Sunnyvale, California, United States). The matrix solution used for MALDI experiments containing αCHCA (α-cyano-4-hydroxycinnamic acid) was from Agilent Technologies (Palo Alto, California, United States). Solvents were purchased from Fisher Chemicals (Tustin, California, United States); all other reagents were obtained from Sigma-Aldrich-Fluka.

#### Enzymatic digestions

The gel bands were cut in a laminar flow hood under conditions to minimize contamination. Sample reduction, alkylation, and digestion was carried out a using Genomic Solutions Proprep digestion robot (Genomic Solutions, Ann Arbor, Michigan, United States). The in-gel digestions were performed according to a modified in-house protocol, under laminar flow. Reduction with 10 mM DTT was allowed to proceed for 20 min at 50 °C. Iodoacetamide was dissolved in 20 mM NH_4_HCO_3_ (pH 8.2) with 10% ACN, and alkylation of cysteine residues was carried out for 1 h at room temperature. Tryptic digestion was initiated by the addition of 1% (w/w) of side-chain-modified, TPCK-treated porcine trypsin and was allowed to proceed at 37 °C for 4 h. The digests were extracted manually with 40 μl of ammonium bicarbonate buffer solution followed by two 30-μl extractions with 60% acetonitrile. The digest extracts were pooled and concentrated to approximately 30 μl, and 10 μl of each mixture was desalted using C18 Zip Tips (Millipore). The samples were eluted into 10 μl (60% ACN, 0.2% TFA), and the volume was reduced to 3 μl using vacuum centrifugation. Targets for MALDI were spotted using the dried-droplet method by adding 0.7 μl of the sample and 1.0 μl of α,CHCA matrix solution. For ESI experiments, the remaining sample (approximately 2 μl) was injected onto a nano-capillary C18 column for HPLC separation.

#### MALDI-TOF/TOF-MS analysis

MALDI-MS data were acquired in an automated mode using a 4700 Proteomics Analyzer (Applied Biosystems, Foster City, California, United States). This instrument employed a neodymium:yttrium aluminum garnet frequency-tripled laser operating at a wavelength of 354 nm and a laser repetition rate of 200 Hz. Initially, a MALDI-MS spectrum was acquired from each spot (1,000 shots/spectrum). Then peaks with a signal to noise ratio greater than 15 in each spectrum were automatically selected for MALDI-CID-MS analysis (7500 shots/spectrum). A collision energy of 1 keV was used with air as the collision gas for collision-induced dissociation (CID) accumulation. After acquisition, the data were subjected to automatic baseline correction, mathematically smoothed, and stored in an Oracle database. Assuming that all ions were singly charged, peak lists from all MS/MS spectra were automatically extracted from the Oracle database and submitted for batch-analysis database searching using an in-house copy of Protein Prospector (version 4.3) with the new program LCBatch-Tag or an in-house copy of Mascot, version 1.8 (Matrix Science, Boston, Massachusetts, United States). The latter was managed using the Mascot Daemon (Matrix Science, Boston, Massachusetts, United States) program running on the same computer. The MS/MS mass values submitted to both search engines were limited using the following criteria: minimum S/N threshold was 8–10, masses of 0–60 Da and within 20 Da of the precursor were excluded, and a maximum of 60 peaks per spectrum were submitted.

Protein Prospector searches were performed by specifying the inclusion of high-energy fragment ions characteristic of the TOF/TOF instrument, whereas Mascot searches included only the low-energy fragment ions and internal ions. For externally calibrated spectra, the allowed mass tolerance that was specified between expected and observed masses for searches was ±75 ppm for MS data, ±200 ppm for MS/MS parent ions, and ±250 ppm for MS/MS fragment ions. All samples were searched against the nonredundant National Center for Biotechnology Information database (NCBInr.02.25.2002).

#### nLC-ESI-Qq-TOF MS analysis

Tryptic peptides were subject to LC-MS/MS analysis on a QSTAR Pulsar mass spectrometer (MDS Sciex, Concord, Ontario, Canada) operating in positive-ion mode. Chromatographic separation of peptides was performed as described earlier except that formic acid was used as the ion-pairing agent. The LC eluent was directed to a micro-ionspray source. Throughout the running of the LC gradient, MS and MS/MS data were recorded continuously based on a 5-s cycle time. Within each cycle, MS data were accumulated for 1 s, followed by CID acquisitions of 4 s on ions selected by preset selection parameters of the information-dependent acquisition (IDA) method. In general the ions selected for CID were the most abundant in the MS spectrum, except that singly charged ions were excluded and dynamic exclusion was employed to prevent repetitive selection of the same ions within a preset time. Collision energies were programmed to be adjusted automatically according to the charge state and mass value of the precursor ions. Peak lists for database searching were created using a script from within the Analyst software. Searches were performed using the two search engines meantioned earlier except that only the low-energy CID fragments characteristic of the ESI-Qq-TOF instrument were considered. The allowed mass-tolerance range between expected and observed masses for searches was ±100 ppm for MS peaks and ±0.1 Da for MS/MS fragment ions.

#### Expression and purification of recombinant CBR1

Isopropyl-β-D-thiogalactoside (final: 1 mM) was added to cultures of E. coli BL21(DE3) harboring pET-11aCR vectors when absorption at 600 nm of the culture became 0.6–0.7 AU. The cells were centrifuged after induction for 4–6 h, then the pellet was suspended in the buffer A and sonicated. The solution was centrifuged for 15 min at 10,000 *g* and 4 °C. The supernatant was loaded on Glutathione Sepharose 4B beads (Amersham Pharmacia Biotech, Little Chalfont, United Kingdom). Beads were washed four times with buffer A. Bead-binding proteins were eluted with 50 mM sodium phosphate (pH 6.1) containing 500 mM NaCl and 20 mM glutathione. The eluate was loaded on PD-10 columns (Amersham Pharmacia Biotech) to exchange the buffer to 10 mM Tris-HCl (pH 7.4).

#### CBR1 assay

CBR1 activity was determined spectrophotometrically at 25 °C. The standard assay mixture consisted of 50 mM sodium phosphate (pH 6.8), 200 μM NADPH, and 200 μM menadione in a total volume of 1 ml. Compounds were dissolved in DMSO as 100× stock solutions. Reactions were initiated by the addition of enzyme, and initial rates were determined by monitoring the disappearance of NADPH at 340 nM. Controls without substrates or enzyme were routinely included.

#### Kinase assays

Fyn and p38 kinases were expressed in bacteria and purified as previously described [13,37]. Protein kinase A and Protein kinase B were obtained commercially (Upstate Cell Signaling Specialties Charlottesville, Virginia, United States). For the inhibition assay, various concentrations of inhibitor were incubated with 50 mM Tris (pH 8.0), 10 mM MgCl_2_, 1.6 mM glutathione, 1 mg/ml BSA, 0.1 mg/ml of the requisite substrate peptide (see Figure S1), 3.3% DMSO, 11 nM (2 μCi) [γ-^32^P]ATP (6,000 Ci/mmol, NEN), and kinase in a total volume of 30 μl for 30 min. Reaction mixtures (27 μl) were spotted onto a phosphocellulose disk and washed with 0.5% H_3_PO_4_. The transfer of ^32^P was measured by standard scintillation counting. The IC_50_ values were defined to be the concentration of inhibitor at which the counts per minute was 50% of the control disk. When the IC_50_ value fell between two measured concentrations, it was calculated based on the assumption of an inversely proportional relationship between inhibitor concentration and counts per minute between the two data points.

#### Cell viability assay and PI-stained cell assay

Cell viability was determined by the alamarBlue reagent reduction assay in a 96-well culture plate, measuring the absorbance at 570 and 600 nm spectrophotometrically. The amount of PI-stained cells was estimated by measuring fluorescence following incubation with PI (5 μg/ml). For both experiments, data are shown as the percentages of nontreated control cells.

#### p53 and ubiquitination detection in A549 cells

A549 cells maintained as described earlier were seeded in 6-cm dishes at a density of 1.8 × 10^4^ cells/cm^2^ and incubated in the presence of 3 ml of F-12K and 10% FBS. After 48 h, the medium was replaced with 3 ml of serum-free F-12K containing either PP2, hydroxy-PP, or hydroxy-PP-Me (prepared from 5 mM DMSO stock solutions), each at concentrations of 2, 5, or 10 μM. Experiments were routinely performed in duplicate, and controls with representative DMSO concentrations were included. After 48 h incubation, the medium was removed, and the cells were lysed with 0.5 ml of modified RIPA buffer (1% NP-40, 50 mM Tris, 150 mM NaCl, 2 mM EDTA, 2 mM Na_3_VO_4_, 0.1% SDS, 0.1 mM DTT, and one Complete Mini, EDTA-free protease inhibitor tablet [Roche, Basel, Switzerland] per 10 ml) for 10 min. The contents of each plate were transferred to 1.5-ml micro-centrifuge tubes, the tubes were sonicated in a bath sonicator for 5 min, and the lysates were cleared by centrifugation (14,000 *g,* 10 min). The supernatant was collected, and the protein concentration was assayed using the Bio-Rad DC protein assay (Bio-Rad, Hercules, California, United States). Equal quantities of protein boiled for 1 min in 1× Lamelli buffer were subjected to electrophoresis using 7.5% acrylamide Tris-Cl Criterion gels (Bio-Rad). Proteins were electrophoretically transferred to nitrocellulose and immunoblotted with DO-1 anti-p53 HRP conjugated antibody or the anti-ubiquitin antibody Ub followed by anti-mouse IgG conjugated HRP (Santa Cruz Biotechnology, Santa Cruz, California, United States). Immuno-reactive proteins were analyzed by enhanced chemiluminescence (Pierce Biotechnology).

#### Protein crystallization

The CBR1 was expressed in E. coli and purified as described earlier. CBR1 (18 mg ml^−1^) in 30 mM sodium phosphate (pH 6.5), 100 mM KCl, and 20 μM DTT was incubated for 30 min with hydroxy-PP (1 mM final concentration) prior to crystallization. Within 2 d at room temperature, good diffracting crystals of the orthorhombic space group P2_1_2_1_2_1_ were obtained by vapor diffusion from 100 mM 2-(N-ethylmorpholino)ethanesulfonate (pH 6.5), 2.0 M ammonium sulfate, and 5% PEG 400.

#### Data collection and structure determination

Orthorhombic crystals of CBR1–hydroxy-PP diffracted to 1.1 Å. A full dataset was collected at the Advanced Light Source (Berkeley, California, United States) beamline 8.3.1 with an ADSC Quantum 4 CCD detector. The dataset was integrated and merged using the HKL2000 and SCALEPACK programs (HKL Research, Charlottesville, Virginia, United States) [37]. The structure was solved by molecular replacement with AMoRe [18] using a modified porcine carbonyl reductase model (1N5D). Crystallographic refinement to 1.2 Å was carried out and electron density maps were produced using SHELXL [20]. Model building was done using O [38] and Quanta 2000 (Molecular Simulations, San Diego, California, United States). Detailed data and refinement statistics are listed in Table S5.

## Supporting Information

Figure S1 Structures of Compounds Included in the Screening Library (2.6 MB PDF).

Figure S2 Immunoblot for CBR1 Expression in the Six Cell Lines Analyzed (554 KB AI).

Figure S3 siRNA Knockdown of p53 and CBR1 during Serum-Withdrawal-Induced Apoptosis (2.3 MB AI).

Figure S4 Synthesis of Hydroxy-PP-Me (518 KB AI).

Table S1 Attributes Used to Characterize Screening Compounds (30 KB DOC).

Table S2 Definition of Components in Figure 1B (19 KB DOC).

Table S3 Inhibition Values of PP, Hydroxy-PP, PP2, and Hydroxy-PP-Me against Four Protein Kinases (30 KB DOC).

Table S4 MS/MS Identification of Affinity-Purified Peptides (31 KB DOC).

Table S5 Data Collection and Refinement (49 KB DOC).

### Accession Numbers

The SwissProt (http://www.ebi.ac.uk/swissprot/) accession numbers for CBR1 and NQO1 are P16152 and P15559, respectively. The ProSite (http://au.expasy.org/prosite/) accession number for human CBR1 is PS00061. The Protein Data Bank (http://www.rcsb.org/pdb/) accession numbers for the other gene products discussed in this paper are porcine carbonyl reductase (PDB 1N5D), Hck (PDB 1QCF), and short-chain dehydrogenase substrate complexes (PDB 2AE2, 1FDS, and 1HZJ). The accession number for the crystal structure of the modified human carbonyl reductase model refined with SHELXL to 1.24 Å with a crystallographic R-factor of 10.3% and a free R-factor of 13.4% is PDB 1WMA.

## Abbreviations

CBR1: carbonyl reductase 1
CID: collision-induced dissociation
DMSO: dimethyl sulfoxide
ESI: electrospray ionization
FBS: fetal bovine serum
hydroxy-PP: 3-(1-*tert*-butyl-4-amino-1H-pyrazolo[3,4-d]pyrimidin-3-yl)phenol
hydroxy-PP-Me: 3-(7-isopropyl-4-(methylamino)-7H-pyrrolo[2,3-d]pyrimidin-5yl)phenol
MALDI: matrix-assisted laser desorption/ionization
MS: mass spectrometry
PCA: principal component analysis
PI: propidium iodide
PP: 1-*tert*-butyl-3-phenyl-1H-pyrazolo[3,4-d]pyrimidin-4-amine
PP1: 1-*tert*-butyl-3-p-tolyl-1H-pyrazolo[3,4-d]pyrimidin-4-amine
PP2: 1-*tert*-butyl-3-(4-chlorophenyl)-1H-pyrazolo[3,4-d]pyrimidin-4-amine
RNAi: RNA interference;
