# Supplementary material for: An Unbiased Cell Morphology–Based Screen for New, Biologically Active Small Molecules
Source: PLoS Biol. 2005 Apr 5;3(5):e128. doi: 10.1371/journal.pbio.0030128 (PMC1073692; doi:10.1371/journal.pbio.0030128)
Supplement: Supplementary file 1 [file pbio.0030128.sg001.pdf]

Supplementary Figure 1. Structures of compounds included in the screening library.

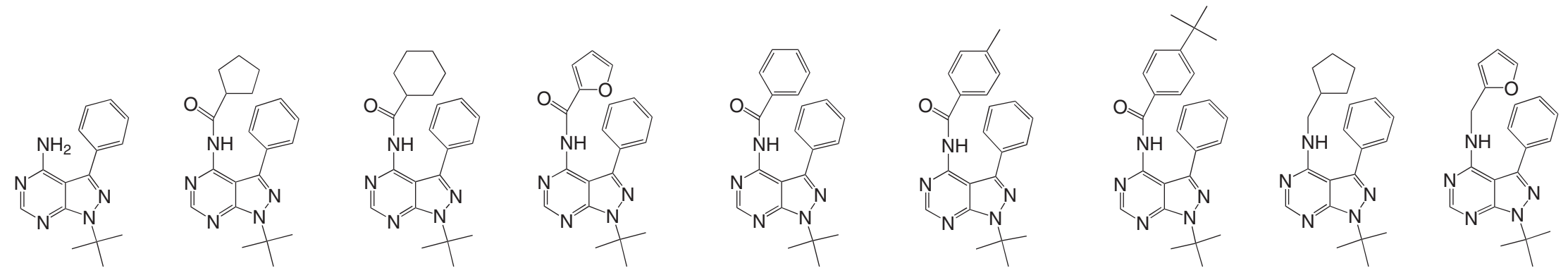

1, AB001; FW: 267.3 **PP** | **2,** AB006; FW: 363.5 | **3,** AB007; FW: 377.5 | **4,** AB008; FW: 361.4 | **5,** AB009; FW: 371.4 | **6,** AB010; FW: 385.5 | **7,** AB011; FW: 427.5 | **8,** AB012; FW: 349.5 | **9,** AB013; FW: 347.4

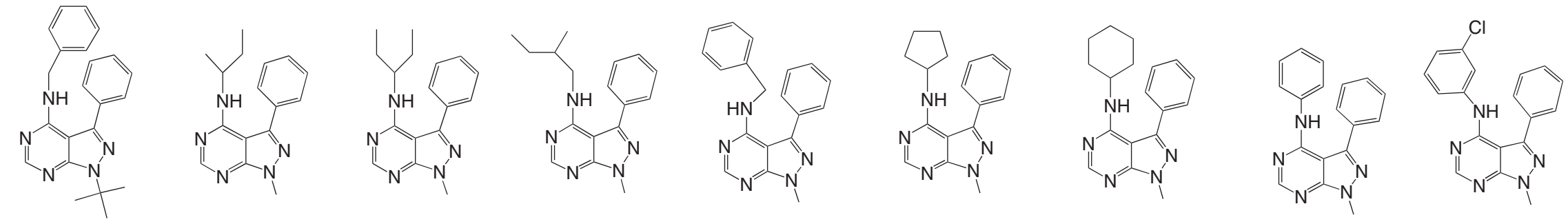

10, AB014; FW: 357.5 | **11,** AB015; FW: 281.4 | **12,** AB016; FW: 295.4 | **13,** AB017; FW: 295.4 | **14,** AB019; FW: 315.4 | **15,** AB020; FW: 293.4 | **16,** AB021; FW: 307.4 | **17,** AB022; FW: 301.4 | **18,** AB023; FW: 335.8

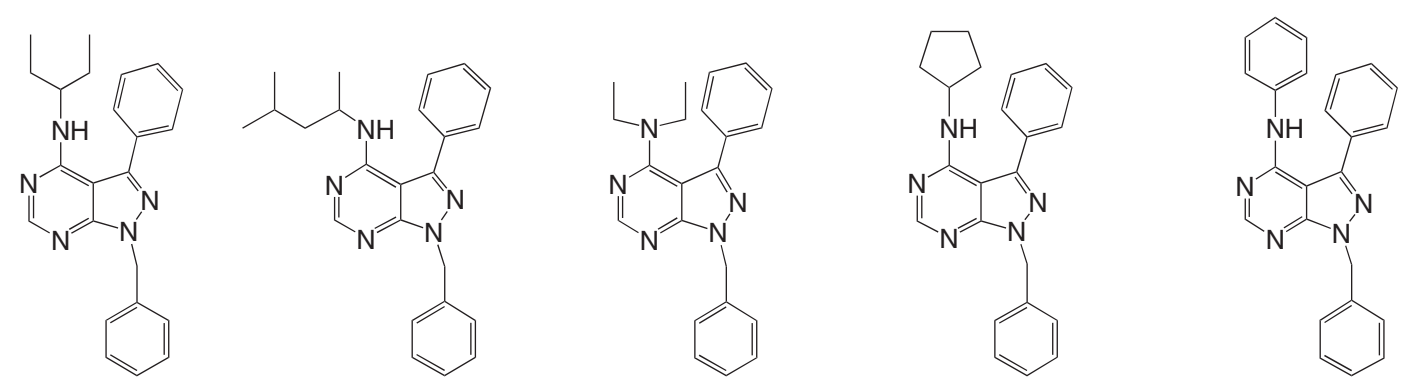

19, AB024; FW: 371.5 | **20,** AB025; FW: 385.5 | **21,** AB026; FW: 357.5 | **22,** AB027; FW: 369.5 | **23,** AB028; FW: 377.4

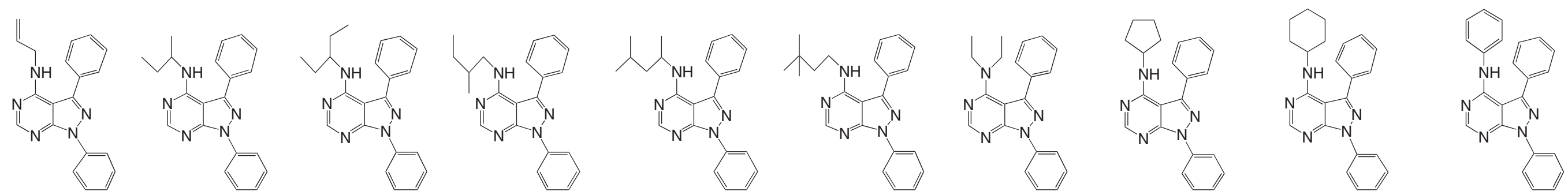

24, AB029; FW: 327.3 | **25,** AB030; FW: 343.4 | **26,** AB031; FW: 357.4 | **27,** AB032; FW: 357.5 | **28,** AB033; FW: 371.5 | **29,** AB034; FW: 371.5 | **30,** AB035; FW: 343.4 | **31,** AB036; FW: 355.4 | **32,** AB037; FW: 369.5 | **33,** AB038; FW: 363.4

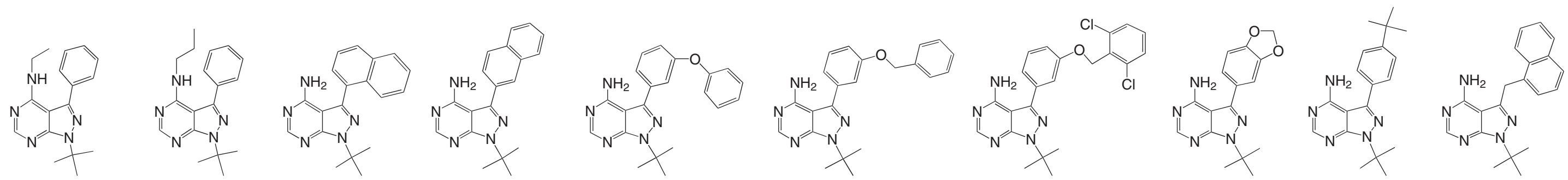

34, AB039; FW: 295.4 | **35,** AB040; FW: 309.4 | **36,** AB041; FW: 317.4 | **37,** AB042; FW: 317.4 | **38,** AB043; FW: 359.4 | **39,** AB044; FW: 373.5 | **40,** AB045; FW: 442.3 | **41,** AB046; FW: 311.3 | **42,** AB047; FW: 323.4 | **43,** AB048; FW: 331.4

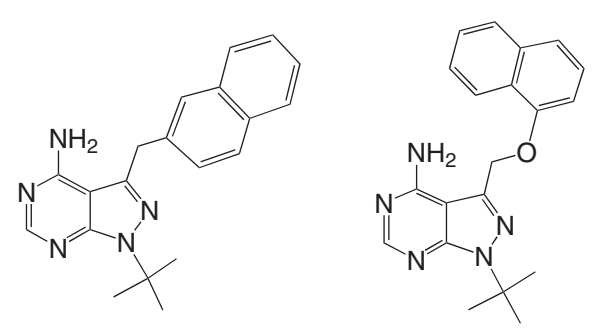

44, AB049; FW: 331.4 | **45,** AB050; FW: 347.4

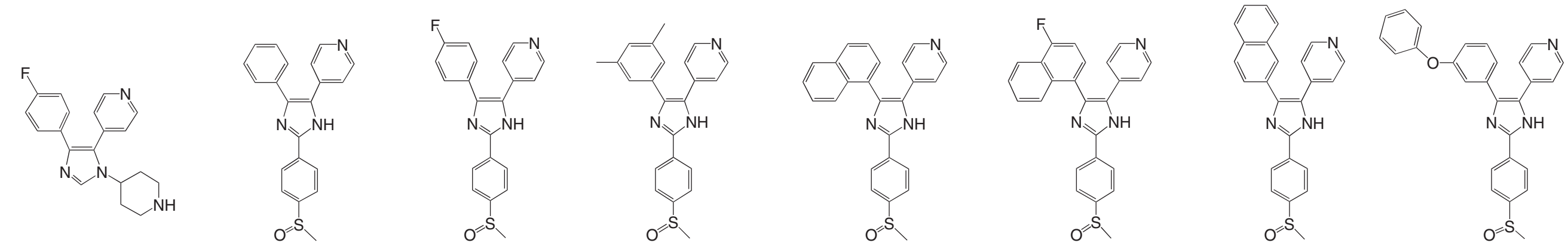

46, VK-19911; FW: 322.4 | **47,** AB051; FW: 359.5 | **48,** AB052; FW: 377.4 | **49,** AB053; FW: 387.5 | **50,** AB054; FW: 409.5 | **51,** AB055; FW: 427.5 | **52,** AB056; FW: 409.5 | **53,** AB057; FW: 451.5

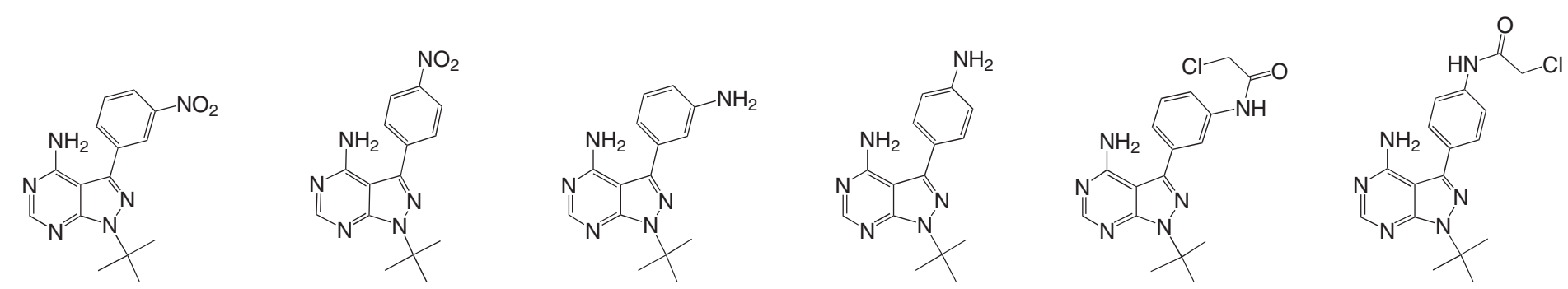

54, AB058; FW: 312.3 | **55,** AB059; FW: 312.3 | **56,** AB060; FW: 282.3 | **57,** AB061; FW: 282.3 | **58,** AB062; FW: 358.8 | **59,** AB063; FW: 358.8

Supplementary Figure 1 (continued). Structures of compounds included in the screening library.

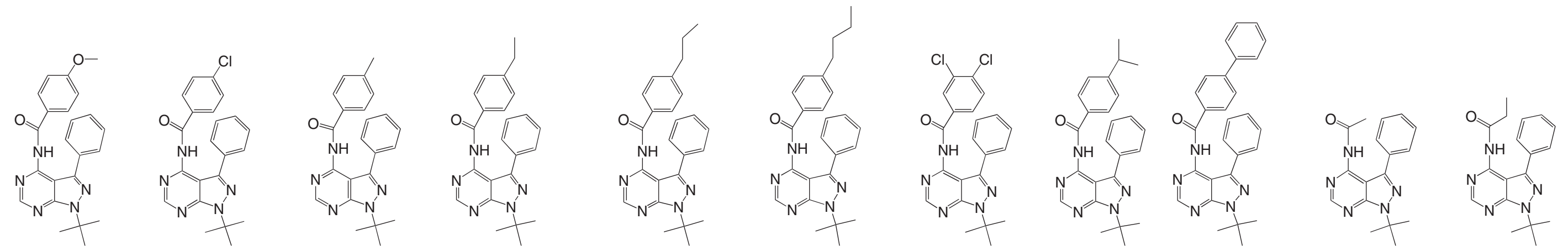

60, AB101; FW: 401.5 **61,** AB102; FW:405.9 **62,** AB103 **63,** AB104; FW: 399.5 **64,** AB105; FW: 413.5 **65,** AB106; FW: 427.5 **66,** AB107; FW: 440.3 **67,** AB108; FW: 413.5 **68,** AB109; FW: 447.5 **69,** AB110; FW: 309.4 **70,** AB111; FW: 323.4

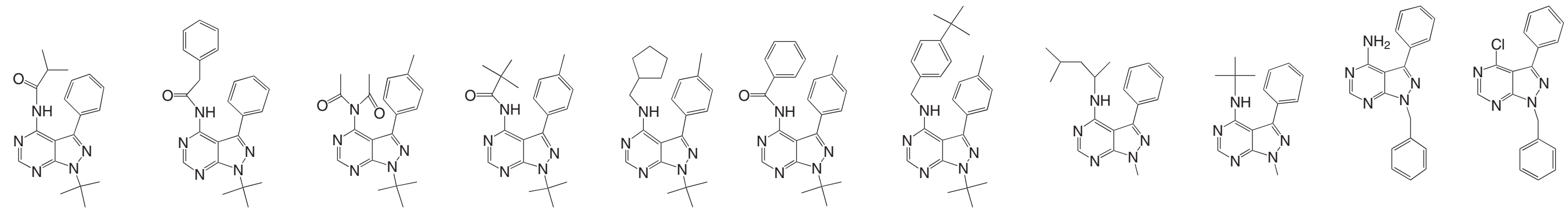

71, AB112; FW: 337.4 **72,** AB114; FW: 385.5 **73,** AB115; FW: 365.4 **74,** AB116; FW: 365.5 **75,** AB117; FW: 363.5 **76,** AB118; FW: 385.5 **77,** AB119; FW: 441.6 **78,** AB120; FW: 309.4 **79,** AB121; FW: 281.4 **80,** AB122; FW: 301.4 **81,** AB123; FW: 320.8

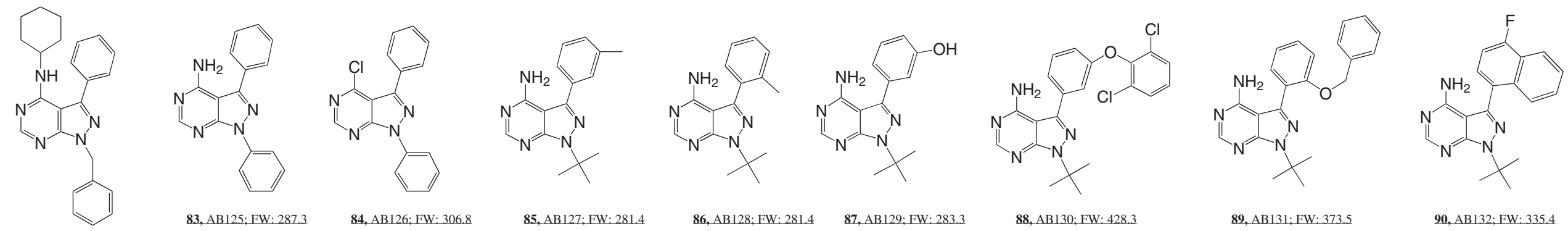

82, AB124 **83,** AB125; FW: 287.3 **84,** AB126; FW: 306.8 **85,** AB127; FW: 281.4 **86,** AB128; FW: 281.4 **87,** AB129; FW: 283.3 **88,** AB130; FW: 428.3 **89,** AB131; FW: 373.5 **90,** AB132; FW: 335.4

Hydroxy-PP

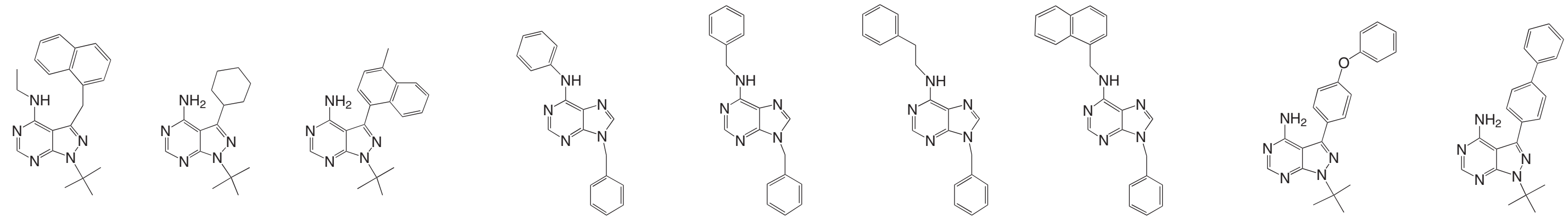

91, AB133; FW: 359.5 **92,** AB134; FW: 273.3 **93,** AB135; FW: 331.3 **94,** SMU1; FW: 301.4 **95,** SMU2; FW: 315.4 **96,** SMU3; FW: 329.4 **97,** SMU4; FW: 365.4 **98,** CZ12; FW: 359.4 **99,** CZ28; FW: 343.4

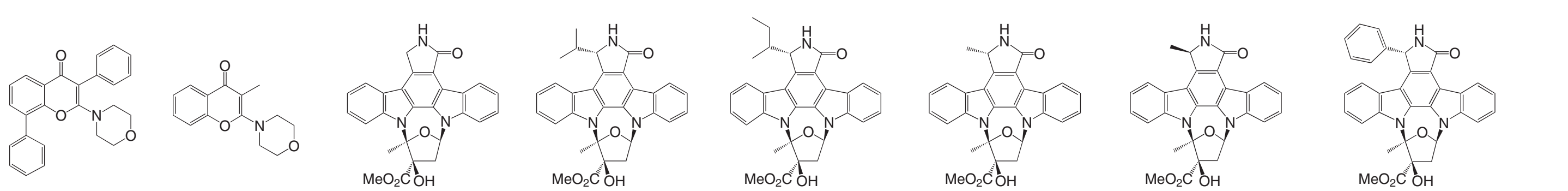

100, PJA-I-107 **101,** PJA-I-117 **102,** (+)K252a; FW: 467.5 **103,** DP001; FW: 509.6 **104,** DP003; FW: 523.6 **105,** DP007; FW: 509.6 **106,** DP008; FW: 509.6 **107,** DP010; FW: 509.6
