# Supplementary material for: An Unbiased Cell Morphology–Based Screen for New, Biologically Active Small Molecules
Source: PLoS Biol. 2005 Apr 5;3(5):e128. doi: 10.1371/journal.pbio.0030128 (PMC1073692; doi:10.1371/journal.pbio.0030128)
Supplement: Supplementary file 2 [file pbio.0030128.st001.doc]

**Supplementary Table 1.** Attributes used to characterize screening compounds.

| **Attribute** | **Description** |
| --- | --- |
| Adhesion coefficient | Relative change in cell counts upon extensive washing of wells |
| Average area, interphase | Average area of interphase cells in a population |
| Average area, mitotic | Average area of mitotic cells in a population |
| Average cell axes ratio, interphase | Average ratio of short axis of an ellipse that has the same second moment as a cell mask to its long axis in interphase cells |
| Average cell axes ratio, mitotic | Average ratio of short axis of an ellipse that has the same second moment as a cell mask to its long axis in all mitotic cells |
| Average cell axes ratio, pre-anaphase | Average ratio of short axis of an ellipse that has the same second moment as a cell mask to its long axis in pre-anaphase mitotic cells |
| Average cell form factor, interphase | Average formfactor (ratio of area to square of perimeter) for all interphase cells in a population |
| Average cell form factor, mitotic | Average formfactor (ratio of area to square of perimeter) for all mitotic cells in a population |
| Average cell form factor, pre-anaphase mitotic | Average formfactor (ratio of area to square of perimeter) for pre-anaphase mitotic cells in a population |
| Average cell moment, interphase | Average moment of masks of all interphase cells in a population |
| Average cell moment, mitotic | Average moment of masks of all mitotic cells in a population |
| Average cell moment, pre-anaphase mitotic | Average moment of masks of all pre-anaphase mitotic cells in a population |
| Average Golgi intensity, interphase | Average of mean Lens Culinaris pixel intensities for all interphase cells in a population |
| Average Golgi intensity, mitotic | Average of mean Lens Culinaris pixel intensities for all mitotic cells in a population |
| Average Golgi kurtosis, interphase | Average kurtosis of Lens Culinaris lectin in interphase cells. |
| Average Golgi kurtosis, mitotic | Average kurtosis of Lens Culinaris lectin in mitotic cells. |
| Average Hoechst intensity, interphase | Average of mean Hoechst pixel intensities within nuclear masks for all interphase cells in a population |
| Average nuclei area, interphase | Average area of nuclei of interphase cells |
| Average nuclei axes ratio, interphase | Average ratio of short axis of an ellipse that has the same second moment as a nuclear mask to its long axis in interphase cells |
| Average total tubulin intensity over DNA mask, mitotic | Average of the sum of Dm1 alpha pixel intensities for all mitotic cells in a population. Only pixels contained within nuclear masks are included in computation |
| Average total tubulin intensity, interphase | Average of the sum of Dm1 alpha pixel intensities for all interphase cells in a population |
| Average total tubulin intensity, mitotic | Average of the sum of Dm1 alpha pixel intensities for all mitotic cells in a population |
| Average total tubulin intensity, pre-anaphase mitotic | Average of the sum of Dm1 alpha pixel intensities for pre-anaphase mitotic cells in a population |
| Average tubulin intensity, interphase | Average of mean Dm1a pixel intensities for all interphase cells in a population |
| Average tubulin kurtosis, interphase | Average kurtosis of Dm1a intensity in interphase cells |
| Average tubulin kurtosis, mitotic | Average kurtosis of Dm1a intensity in mitotic cells |
| Average tubulin kurtosis, pre-anaphase mitotic | Average kurtosis of Dm1a intensity in pre-anaphase mitotic cells |
| G1 proportion | Proportion of cells in G1 phase of cell cycle in a population |
| G2 proportion | Proportion of cells in G2 phase of cell cycle in a population |
| Mitotic index | Proportion of mitotic cells in a population |
| Post-anaphse proportion | Proportion of post-anaphase mitotic cells in a population |
| Pre-anaphase proportion | Proporion of pre-anaphase mitotic cells in a population |
| Proportion of "branched" cells, interphase | Proportion of interphase cells with 3 or more well formed projections in a population of all cells |
| Proportion of "branched" cells, mitotic | Proportion of mitotic cells with 3 or more well formed projections in a population of all cells |
| Relative number of cells | Relative number of cells with respect to untreated cells |
| S proportion | Proportion of cells in S-phase of cell cycle in a population |
| STD of Hoechst intensity, interphase | Average standard deviation of Hoechst pixel intensities within nuclear masks for all interphase cells in a population |
| STD of tubulin intensity, interphase | Average standard deviation of Dm1 alpha pixel intensities for all interphase cells in a population |
