# Supplementary material for: An Unbiased Cell Morphology–Based Screen for New, Biologically Active Small Molecules
Source: PLoS Biol. 2005 Apr 5;3(5):e128. doi: 10.1371/journal.pbio.0030128 (PMC1073692; doi:10.1371/journal.pbio.0030128)
Supplement: Supplementary file 3 [file pbio.0030128.st002.doc]

**Supplementary Table 2:** Definition of components in Figure 1B.

**Component	Feature	Weighting**

Comp. 1:	G2.MIT.PROP.Tubulin.SF268	0.177791

Comp. 1:	MITOTIC.INDEX.Tubulin.SF268	0.17309

Comp. 1:	MITOTIC.INDEX.Tubulin.SKOV3	0.169122

Comp. 1:	AVG.AXES.RATIO.INT.Tubulin.SF268	-0.166

Comp. 1:	AVG.AREA.INT.Tubulin.SF268	-0.16533

Comp. 2:	G1.MIT.PROP. Tubulin.A549	-0.23375

Comp. 2:	DM1A.STD.DD2.Lectin.SKOV3	0.211289

Comp. 2:	G1.MIT.PROP.Tubulin.HUVEC	-0.20356

Comp. 2:	S.INT.PROP.Tubulin.A549	0.200519

Comp. 2:	G2.MIT.PROP.Tubulin.SKOV3	0.198568

Comp. 3:	AVG.AREA.INT.Tubulin.A549	-0.33667

Comp. 3:	G2.INT.PROP.Tubulin.A549	-0.31407

Comp. 3:	G2.INT.PROP.Tubulin.SKOV3	-0.26149

Comp. 3:	G2.INT.PROP.Tubulin.DU-145	-0.22287

Comp. 3:	G1.MIT.PROP.Tubulin.A549	0.211219
