# Supplementary material for: An Unbiased Cell Morphology–Based Screen for New, Biologically Active Small Molecules
Source: PLoS Biol. 2005 Apr 5;3(5):e128. doi: 10.1371/journal.pbio.0030128 (PMC1073692; doi:10.1371/journal.pbio.0030128)
Supplement: Supplementary file 4 [file pbio.0030128.st003.doc]

**Supplementary Table 3**. Inhibition of PP, Hydroxy-PP, PP2 and Hydroxy-PP-Me against four protein kinases.

|  | **IC_50_ (nM)** | | **Relative activity @ 1 µM inhibitor** | |
| --- | --- | --- | --- | --- |
|  | **Fyn^a^** | **p38^b^** | **PKA^c^** | **PKB^d^** |
| **PP** | **110** | **4,000** | **92%** | **92%** |
| **Hydroxy-PP** | **8** | **17,000** | **110%** | **91%** |
| **PP2** | **50** | **900** | **96%** | **103%** |
| **Hydroxy-PP-Me** | **> 50,000** | **> 50,000** | **106%** | **95%** |

IC_50_ values against Fyn and p38 are given (left), and percentage activity of PKA and PKB in presence of the inhibitors are given (right). Kinase activity was determined using a standardized disk assay based on the P^32^ transfer to a synthetic peptide (See Materials and Methods). Kinase reactions were run for 25 min in presence of 11 nM (2 µCi) [γ-^32^P]ATP. Peptide subrate sequences for each kinase are shown below:

a. LEEIYGEFKKK (100 µM) was used as the substrate for Fyn.
b. IPTSPITTTYFFFKKK (100 µM) was used as the substrate for p38.
c. bzRRASLG (100 µM) was used as the susbtrate for PKA.
d. bzRPRTSSF (100 µM) was used as the susbtrate for PKB.
