# Supplementary material for: An Unbiased Cell Morphology–Based Screen for New, Biologically Active Small Molecules
Source: PLoS Biol. 2005 Apr 5;3(5):e128. doi: 10.1371/journal.pbio.0030128 (PMC1073692; doi:10.1371/journal.pbio.0030128)
Supplement: Supplementary file 5 [file pbio.0030128.st004.doc]

**Supplementary Table 4.** MS/MS identification of affinity purified proteins.

| **Protein Identification Results** | | |  |  |
| --- | --- | --- | --- | --- |
| Band Number | Accession # | Name of Protein ID | MALDI (TOF/TOF) ID | ESI (Qq-TOF) ID |
| A1 & A2 | 4505701 | Pyridoxal Kinase | x | x |
| B1, B2 & B3 | 4502599 | Carbonyl Reductase 1 | x | x |
| C1 | 4505409 | Nm23 protein | - | x |
| C2* | - | - | - | - |
| C3 | 1421609 | Nucleoside Diphosphate Kinase | - | x |

* Only trypsin autolysis products were detected.

*MALDI TOF/TOF and ESI Q-qTOF*

- No carry over from Fetuin A, bradykinin, or the previous days runs were observed.
- For database searches all species were included.
- NCBInr database was used for all searches.
- All searches were preformed with a mass tolerance of 100 ppm or below.
- Trypsin autolysis products were detected in all experiments and used for internal calibration.
