# Supplementary material for: An Unbiased Cell Morphology–Based Screen for New, Biologically Active Small Molecules
Source: PLoS Biol. 2005 Apr 5;3(5):e128. doi: 10.1371/journal.pbio.0030128 (PMC1073692; doi:10.1371/journal.pbio.0030128)
Supplement: Supplementary file 6 [file pbio.0030128.st005.doc]

**Supplementary Table 5.** Data Collection and Refinement Statistics.

|  | | CBR1-Hydroxy-PP |
| --- | --- | --- |
|  | |  |
| Space group | | P2_1_2_1_2_1_ |
| Cell constants (Å) | | 54.5 55.4 95.9 |
| Observed/ unique reflections | | 316213/155494 (10 – 1.24 Å) |
| Completeness | | 99.5% (96.0% 1.28 – 1.24 Å) |
| R_sym_ | | 3.7% (12.6% 1.28 – 1.24 Å) |
| Refinement | |  |
|  | Number of non-hydrogen atoms per asymmetric unit: | |
|  | Protein | 2229 |
|  | Solvent | 394 |
|  | Heterogen | 163 |
|  | rms deviation |  |
|  | Bond length^a^ (Å) | 0.014 |
|  | Angle distance^a^ (Å) | 0.030 |
|  | R_work_ | 10.3% |
|  | R_free_ | 13.4% |
| ^a^ As calculated by SHELXL $R_{sym}=\sum\left\vert I-\left\langle I\right\rangle\right\vert/\sum I$ | |  |
